# Design, Synthesis and SAR in 2,4,7-Trisubstituted Pyrido[3,2-*d*]Pyrimidine Series as Novel PI3K/mTOR Inhibitors

**DOI:** 10.3390/molecules26175349

**Published:** 2021-09-02

**Authors:** Frédéric Buron, Nuno Rodrigues, Thibault Saurat, Marie Aude Hiebel, Stéphane Bourg, Pascal Bonnet, Reine Nehmé, Philippe Morin, Nathalie Percina, Justine Corret, Béatrice Vallée, Remy le Guevel, Marie-Lise Jourdan, Hélène Bénédetti, Sylvain Routier

**Affiliations:** 1 Institut de Chimie Organique et Analytique, Université d’Orléans, UMR CNRS 7311, rue de Chartres, BP 6759, 45067 Orléans, France; frederic.buron@univ-orleans.fr (F.B.); nuno.rodrigues@univ-orleans.fr (N.R.); thibault.saurat@univ-orleans.fr (T.S.); marie-aude.hiebel@univ-orleans.fr (M.A.H.); stephane.bourg@univ-oleans.fr (S.B.); pascal.bonnet@univ-orleans.fr (P.B.); reine.nehme@univ-orleans.fr (R.N.); philippe.morin@univ-orleans.fr (P.M.); nathalie.percina@univ-orleans.fr (N.P.); 2 Centre de Biophysique Moléculaire, CNRS Orléans, Rue Charles Sadron, 45071 Orléans, France; justine.corretgiudei@cnrs-orleans.fr (J.C.); beatrice.vallee@cnrs.fr (B.V.); 3 Campus de Villejean, ImPACcell, Structure Fédérative de Recherche BIOSIT, Université de Rennes 1, Bat 8, 2 Avenue du Pr. Leon Bernard, CS34317, 35043 Rennes, France; remy.leguevel@univ-rennes1.fr; 4 Nutrition Croissance et Métabolisme, N2C, INSERM U1069, CHU Tours, Faculté de Médecine, 10 boulevard Tonnellé, 37032 Tours, France; m.jourdan@chu-tours.fr

**Keywords:** pyridopyrimidine, synthesis, PI3K and mTOR kinase inhibition, molecular modeling, cell effects

## Abstract

This work describes the synthesis, enzymatic activities on PI3K and mTOR, in silico docking and cellular activities of various uncommon 2,4,7 trisubstituted pyrido[3,2-*d*]pyrimidines. The series synthesized offers a chemical diversity in C-7 whereas C-2 (3-hydroxyphenyl) and C-4 groups (morpholine) remain unchanged, in order to provide a better understanding of the molecular determinants of PI3K selectivity or dual activity on PI3K and mTOR. Some C-7 substituents were shown to improve the efficiency on kinases compared to the 2,4-di-substituted pyrimidopyrimidine derivatives used as references. Six novel derivatives possess IC_50_ values on PI3Kα between 3 and 10 nM. The compounds with the best efficiencies on PI3K and mTOR induced micromolar cytotoxicity on cancer cell lines possessing an overactivated PI3K pathway.

## 1. Introduction

The phosphatidylinositol 3-kinase (PI3K) pathway controls cell proliferation, growth, differentiation, protein synthesis, glucose metabolism, migration, and apoptosis [1,2]. Its activation is initiated by the binding of the corresponding ligands to tyrosine kinase receptors and G-protein coupled receptors (GPCRs). This results in phosphorylation of a regulatory subunit of PI3K (the first enzyme of the pathway) and the subsequent activation of p110, a catalytic subunit of PI3K. This activation leads to the production of phosphatidylinositol 3,4,5-triphosphate (PIP3), a lipid second messenger, at the plasma membrane. PIP3 levels are negatively regulated by phosphatase and tensin homologue (PTEN). PIP3 allows the recruitment of Akt at the membrane through its pleckstrin homology domain (PH). Akt is activated by phosphorylation at the plasma membrane [3,4,5].

Once activated, signaling through Akt propagates to a diverse array of substrates, including the mammalian target of rapamycin (mTOR), a key regulator of protein translation. mTOR is a serine/threonine protein kinase that interacts with several proteins, forming two distinct complexes named mTOR complex 1 (mTORC1) and 2 (mTORC2), which regulate different cellular processes including metabolism, growth, proliferation, and survival [6,7,8,9]. Moreover, activation of the pathway is not vertical, and it has been shown that mTOR exerts a negative feedback loop on PI3K: mTOR once activated will inhibit PI3K through IRS-1 (Insulin Receptor Substrate-1) [10,11]. Furthermore, it has been observed in several studies that external factors (growth factors, nutrient intake) can activate Akt and mTOR, thereby bypassing PI3K and thus restarting cancer genesis [12,13,14,15].

The PI3K pathway is one of the most commonly activated signaling pathways in diverse cancer types, resulting in an extended survival growth and angiogenesis of tumor cells [16,17,18]. In a retrospective analysis of 19′784 patients, Millis et al. identified aberrations in the PI3K/Akt/mTOR pathway in 38% of the solid tumors histologically analyzed; 30% correspond to PTEN loss and 13%, 6% and 1% to mutations in PI3KCA (PI3Kα), PTEN and Akt1, respectively [19].

For these reasons, the need to develop compounds that can inhibit the PI3K pathway has been a great motivation for research teams around the world. Currently developed PI3K pathway inhibitors can be divided into five classes: (i) mTOR inhibitors, (ii) pan-class I PI3K inhibitors, (iii) dual PI3K/mTOR inhibitors, (iv) isoform selective PI3K inhibitors and (v) Akt inhibitors [20,21,22,23,24]. Pharmaceutical companies and academic institutes accomplish significant efforts in the clinical development of PI3K pathway inhibitors for solid tumor treatment, including exploring effective combinations, predictive biomarkers, target patient populations, as well as underlying resistance mechanisms. To date, 126 clinical trials are currently ongoing using Akt inhibitors (including 10 phase III trials) and 140 clinical trials (including 14 phase III) are currently being conducted on mTOR inhibitors either as monotherapy or as part of a combination therapy for many cancer types. For instance, 235 clinical trials are under way on PI3K inhibitors (including 30 phase III) and concern mainly lymphoma and solid tumors.

**Figure 1:**
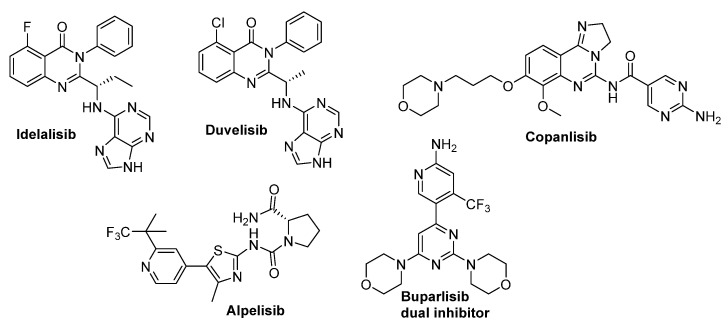
Approved FDA PI3K inhibitors and PI3K/mTOR dual inhibitors in phase III clinical trials.

Currently, two drugs inhibiting the mTOR signaling pathway (Temserolimus and Everolimus) through the binding of FKBP-12 have been approved by the FDA (Food and Drug Administration) for cancer treatment (advanced renal cell carcinoma, tuberous sclerosis) [25,26]. Four PI3K inhibitors have also been FDA-approved (Figure 1): the two benzopyrimidinones idelalisib and duvelisib (PI3K δ and γ-selective) for three types of blood cancer hematologic malignancy or chronic lymphocytic leukemia, respectively [27,28] as well as the tricyclic heterocyclic copanlisib (PI3K α and δ-selective) for follicular lymphoma [29]. Finally, the thiazole derivative, alpelisib, was approved for PIK3CA-mutated advanced breast cancer treatment [24,30].

The mTOR inhibitor effects as cancer monotherapy have been limited, perhaps because they only exhibit poor proapoptotic activity, being mainly cytostatic and because of the existence of a negative feedback loop on PI3K/Akt, resulting in enhanced PI3K/Akt upon mTOR inhibition. Additionally, selective PI3K and Akt inhibitors might not be sufficient to block the entire pathway because of the possible independent activation of mTOR. Therefore, dual PI3K/mTOR inhibitors with pan class I PI3K and mTOR inhibition combine multiple therapeutic efficiencies in a single molecule. They reduce the risk of drug resistance development and prevent PI3K/Akt reactivation due to the negative feedback loop. Due to the awareness of the importance of the retroactive loop, only early phases of clinical trials (25) are found on dual PI3K/mTOR inhibitors and one of them includes morpholinylpyrimidine buparlisib (BKM120 in phase III for metastatic breast cancer) [31].

**Figure 2:**
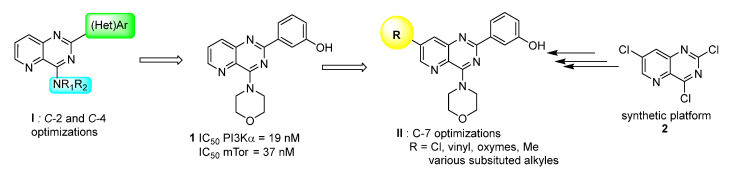
Modulations of C-7 position in the pyrido[3,2-*d*]pyrimidine series.

In this context, we have previously generated a library of *C*-2,4 di-substituted pyridopyrimidines **I**, that were screened for their dual PI3K/mTOR inhibition. Some derivatives are active in the nanomolar range on both enzymatic targets and two of them are highly potent on cancer cells in the submicromolar range without any toxicity on healthy cells. In order to explore the molecular interactions in the active sites of the two kinases and to understand the structural elements leading to selectivity against PI3K or duality against both PI3K and mTOR in this novel chemical series, we focused our efforts on C-2,4,7 tri-substituted pyridopyrimidines of type **II** (Figure 2). This series will offer chemical diversity in C-7 whereas the 2-(3-hydroxyphenyl) and C-4-morpholine groups remained unchanged, because these two pharmacophoric substituents confer a dual inhibition potency on **1**.

In this work, we first established the straightforward pathways able to provide various inventive trisubstituted pyridopyrimidines **II** from a unique synthetic trichlorinated skeleton **2**. Molecular diversity exploration in C-7 position was highlighted by the introduction of vinyl, oximes, chlorine, as well as methyl and variously substituted methylene groups (alcohols, ethers, amines, triazoles and oxazoles) which could become, after SAR analysis, key elements in a better understanding of the drug kinase interactions. With this aim in mind, each final compound was evaluated on the one hand on PI3Kα and mTOR targets in vitro, and on a representative cancer cell line panel. The ability of each compound to inhibit PI3K in cells was checked on one of these cell lines by evaluating the amount of p-Akt by Western blot. In silico docking studies were used to support medicinal chemistry efforts and proved to be successful in explaining the SAR of inhibitors.

## 2. Results

### 2.1. Synthesis

**Scheme 1:**
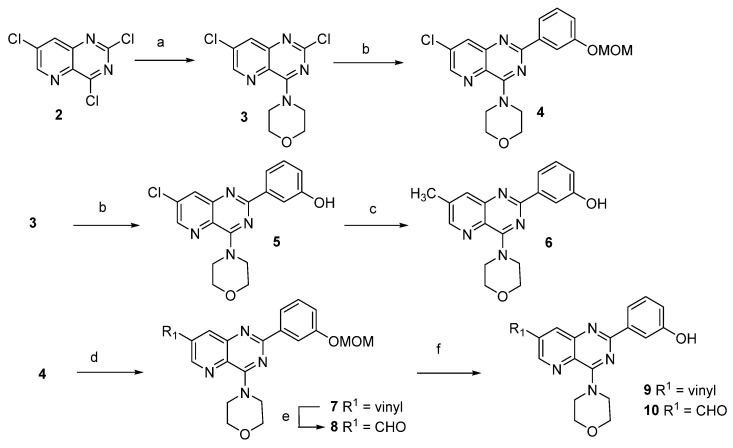
Reagents and conditions: (a) morpholine (1.1 eq.), Et_3_N (1.1 eq.), THF, r.t., 12 h, 91%; (b) 3-methoxymethoxyphenyltrifloroborate potassium salt or 3-hydroxyboronic acid, K_2_CO_3_, Pd(PPh_3_)_4_, DME, μW, 150 °C, 1 h, for **4** or **5**: 71%; (c) AlMe_3_, Pd(PPh_3_)_4_, THF, 70 °C, 16 h, 70%; (d) 4.4.6-trimethyl-2-vinyl-1.3,2-dioxaborolane, K_2_CO_3_, Pd(PPh_3_)_4_, toluene/EtOH, μW, 150 °C, 1 h, 91%; (e) NaIO_4_, OsO_4_, THF/water, r.t., 2 h, 98%; (f) HCl 4 M, dioxane, r.t., 6 h, for **9**: 98%, **10**: 90%.

To explore the molecular diversity in C-7 position, derivative **4** was synthesized after two steps from the 2,4,7-trichloropyrido 3,2-*d* pyrimidine **2** [32]. Condensation of morpholine under S*_N_*Ar first selectively occurred in *C*-4 position. Next a *C*-2 regio-specific arylation with the homemade 3-methoxymethoxyphenyltrifloroborate potassium salt or with 3-hydroxyphenyl boronic acid led, under microwave irradiation, to compounds **4** and **5** in satisfying yields (Scheme 1) [33,34]. The last palladium cross coupling reactions concerned the use of **5** in a *C*-7 methylation involving AlMe_3_ to generate **6** in a 70% yield, and the use of derivative **4** in very efficient vinylation under microwave irradiation to give **7** [32]. To be able to introduce diversity on the *C*-7 position, we prepared the aldehyde **8** using Lemieux–Johnson oxidation conditions (NaIO_4_ and OsO_4_) from **4** in a near quantitative manner. Finally, the MOM protective group removal of **7** and **8** with HCl (4M in dioxane) led to **9** and **10** in excellent yields.

**Scheme 2:**
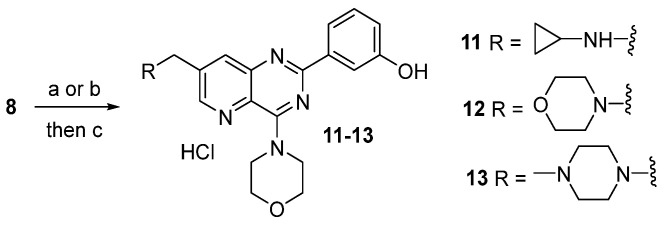
Reagents and conditions: (a) primary amine, NaBH_3_CN, CH_2_Cl_2_, r.t., 12 h; (b) secondary amine, NaBH(OAc)_3_, CH_2_Cl_2_/DMF, acetic acid, r.t., 5 h; (c) HCl 4 M, dioxane, r.t., 6 h, for **11**: 40%, for **12**: 61%, for **13**: 91%.

Reductive aminations were performed on **8** in the presence of cyclic primary or secondary amines and NaBH(OAc)_3_ or NaBH_3_CN as hydride sources, respectively (Scheme 2). The small *C*-7 methyleneaminoalkyl library was obtained with yields ranging between 40 and 91%. Noteworthy, each derivative was isolated as a chlorhydrate salt as the MOM protective groups were directly removed after the reductive amination by acidic hydrolysis.

**Scheme 3:**
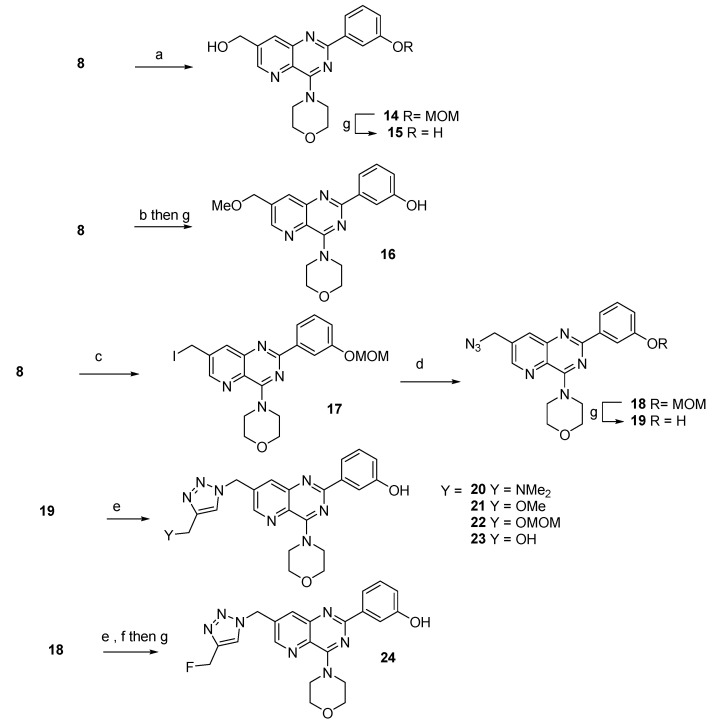
Reagents and conditions: (a) NaBH_4_, MeOH, r.t., 2h, 99%; (b) NaBH_4_, THF then MeI, r.t., 2h30; (c) PPh_3_, I_2_, imidazole, CH_2_Cl_2_, 0 °C, 7 h, 69%; (d) NaN_3_, DMF, 65 °C, 6h, 75%; (e) alkyne, Et_3_N, CH_3_CN, 12 h, r.t., Cu(OAc)_2_ or CuI, for **20**: 31%, for **21**: 79%, for **22**: 25%, for **23**: 51%; (f) DAST, CH_2_Cl_2_, 0 °C, 1h; (g) HCl 4 M, dioxane, r.t., 6 h, for **15**: 87%, for **19**: 74%, for **22**: 98% for **24**: 62%.

Classical aldehyde reduction of **8** gave access to primary alcohol **14** in a quantitative yield (Scheme 3). Williamson methylation and MOM deprotection led to ether **16** in satisfying yield. Additionally, primary alcohol was iodinated and next transformed in azido **18** whereas its MOM deprotection led to **19**. Triazole moieties were generated via Huisgen 1,3-dipolar cycloaddition starting from **19** and using several commercially available or homemade terminal propargylic alkynes [35]. The copper source was adapted as a function of the reactivity and the attempted products **20**–**23** were synthesized in moderate to excellent yields. Treatment of alcohol **23** with DAST failed but the same fluorination method was successfully employed from protecting compound **22**, leading to compound **24** in a good yield after phenol deprotection.

**Scheme 4:**
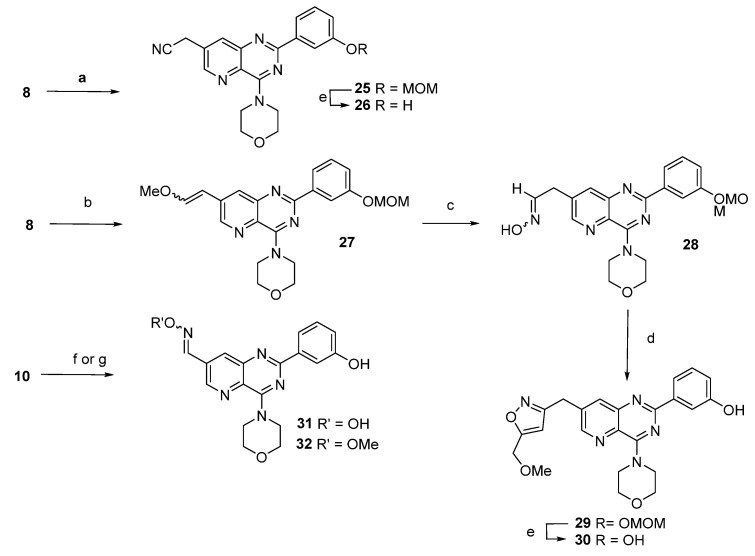
Reagents and conditions: (a) TosMIC, *t*-BuOK, DME, −50 °C, 40 min then MeOH, reflux, 1 h, 46%; (b) PPh_3_CH_2_OMe, *t*-BuOK, THF, 48 h, r.t., 80%; (c) Hg(OAc)_2_, THF/H_2_O, 2h, 0 °C, 90% then NH_2_OH, HCl, Et_3_N, CH_2_Cl_2_, r.t., 12 h, 45%; (d) methyl propargyl ether, NaOCl, THF, 12h, r.t., 50%; (e) HCl 4M, dioxane, 4 h, r.t., for **26**: 84%, for **30**: 98%; (f), Et_3_N, CH_2_Cl_2_, r.t., 12 h, for **31**: 62% with NH_2_OH, HCl; for **32**: 68% with NH_2_OCH_3_, HCl.

Aldehyde **8** provided the cyanomethyl derivative **26** using a small excess of TosMIC in presence of *t*-BuOK, followed by a hydrolysis in acidic media. Next, Oximes **31** and **32** were next straightforwardly prepared using **10** with the adapted hydroxylamine. To finish, the side chain homologation of **6** was carried out using a Wittig/oxidative cleavage procedure and crude aldehyde furnished the oxime **28** in moderate yield. The presence of **28** gave us the opportunity to build the oxazolic derivative **30**, which could be considered as a direct isoster of **21** (Scheme 4).

### 2.2. Kinase Assays

In vitro activities toward the PI3Kα isoform were measured using the final phenolic compounds and IC_50_ values (Table 1) were compared to **1** which inhibits PI3Kα and mTOR with IC_50_ = 19 and 37 nM, respectively. Considering the PI3K target, introduction of a *C*-7 substitution had clearly an influence on the observed kinase activity. A chlorine or methyl group led to derivatives **5** and **6** that are slightly more active than **1** (entries 1, 2). Comparatively, a hydroxymethyl group (**15**, entry 7) led to a significant 30-fold reduction in the inhibition level as if a donor/acceptor hydrogen (DAH) group in this position was not tolerated. The introduction of vinyl, methoxymethyl and cyanomethyl groups partially restored the activity of the corresponding derivatives (entries 3, 8, 14). Finally, the zwitterionic azido derivative **19** exhibited a spectacular IC_50_ of 10 nM (entry 9). Even if they exist as an isomer mixture and could be considered as unstable in living cells, oximes were evaluated and as attempted the hydroxyloxime function of **31** was less tolerated than its more hydrophobic methylated analogue **32** (entries 16, 17), which inhibited the enzyme with an excellent 3 nM IC_50_ value.

The improved activity for several compounds containing relatively remote groups such as methoxyether, vinyl, azido and methyl oxime groups in C-7 could be indicative of the stabilization of molecules with residues located in an aprotic but polar region. With this idea in mind, we decided to increase the size of the C-7 appendix, introducing small and large amines on the C-7 sp^3^ methylene. When amino residues were cyclopropylamine or *N*-methylpiperazine (entries 4, 6), enzymatic inhibition activity took place in the sub-micromolar range. The best result was obtained with the morpholine group as **12** exhibits an IC_50_ = 43 nM. Since substituents containing several polyheteroatomic functions such as azides and oximes had shown a promising inhibitory potency, we next performed the biological evaluation of several azole derivatives.

Triazole substitutions confirmed the previous observation that strong electrically rich hydroxyl or fluorine groups are not well tolerated (entries 12, 13) compared to the presence of the more hydrophobic dimethylamino or methoxymethyl residues (entries 10, 11) of **20** and **21**, which became one of the most active compounds against PI3K with a stable C-7 substituent (IC_50_ = 10 nM). Finally, switching from a triazole to an oxazole ring did not bring additional inhibition potency (entry 15).

As mTOR belongs to the same kinase family as PI3K and displays structural similarities, we next examined the inhibitory activity on mTOR of molecules possessing an IC_50_ on PI3K lower than that measured for **1** (i.e., IC_50_ PI3K = 19 nM). As attempted, all the selected 2,4,7-trisubstituted pyrido[3,2-*d*]pyrimidine compounds displayed diverse degrees of activity against both mTOR and PI3K (Table 2).

It appears immediately that the introduction of a substituent in C-7 decreases the inhibition of mTOR. Nevertheless, the selected derivatives remain active against this kinase and offer IC_50_ around 100 nM. Moreover, IC_50_ measurements showed that chemical modifications based on **1** offered a quasi-selective PI3K inhibitor **32**. Additionally, we increased the number of potent dual inhibitors such as **5**, **19** and **21**, which possess a similar selectivity index (SI) around 10 value. With these three compounds, our aim of improving the inhibition efficiency against PI3k with a C-7 substituent with a moderate impact on the duality of inhibition is achieved. Finally, the two dual derivatives **6** and **21** were chosen to measure their selectivity against the other PI3K isoforms. Values show a very important selectivity for PI3Kα versus other isoforms except for derivative **21** which led to a good additional activity on isoform β. Addition of the triazole ring in C-7 might therefore modify the interaction of the molecule with this isoform.

### 2.3. Docking Studies

To investigate the binding mode of the ligands and analyze the substituent effects on the inhibition and selectivity against PI3Kα and mTOR, we carried out docking studies. The structures of mTOR [36] and PI3Kα [37] were retrieved from the Protein Data Bank (PDB) [38]. Both structures interact with the pyridinylfuranopyrimidine inhibitor PI-103 that contains a morpholine group binding to the hinge through a hydrogen bond. The hydroxy moiety of the phenol is forming a hydrogen bond donor to the carboxylic acid group of Asp2195 side chain at the back of the inner pocket of PI3K. The PI3Kα and mTOR aligned structures were extracted from the MOE kinase-ligand complex library [39]. As stressed by Bryant et al. [40] or Wright et al. [41], the two targets share a high structure similarity but a low sequence identity.

**Figure 3:**
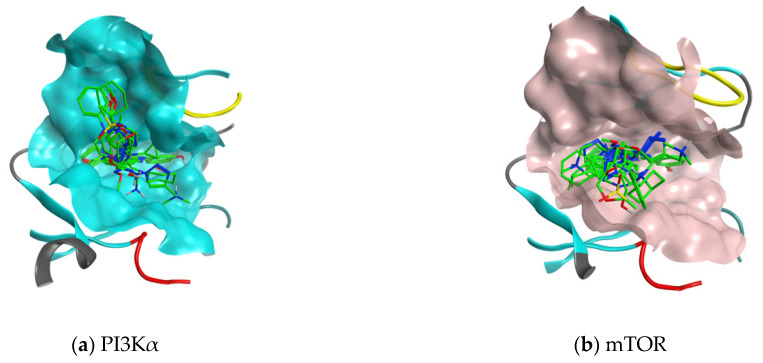
Superimposition of all the best docking poses of all compounds in PI3Kα (**a**) and mTOR (**b**).

All the compounds were docked into the active site of each studied protein, PI3Kα and mTOR. All the compounds exhibited a similar binding mode in which the pyridopyrimidine scaffold of all the docked compounds was well superimposed, creating similar interactions with each active site of the proteins. Only compound **6** has the flipped hydroxyphenyl ring in PI3Kα. As suggested before, the side chains at the *C*-7 position point towards the solvent area and more precisely to the glycine rich loop for PI3Kα (Figure 3a) and the activation loop for mTOR (Figure 3b).

Analysis of the intermolecular interactions between the compounds and residues of the active site clearly showed that the oxygen atom of the morpholine moiety forms a hydrogen bond with Val851 of PI3Kα and Val2240 of mTOR located in the hinge region. The pyridopyrimidine scaffold is positioned in the ATP active site and the hydroxy group of the phenol moiety at the C-2 position is forming a hydrogen bond donor with the carboxylic acid group of Asp810 and Asp2195 residues of PI3Kα and mTOR, respectively.

**Figure 4:**
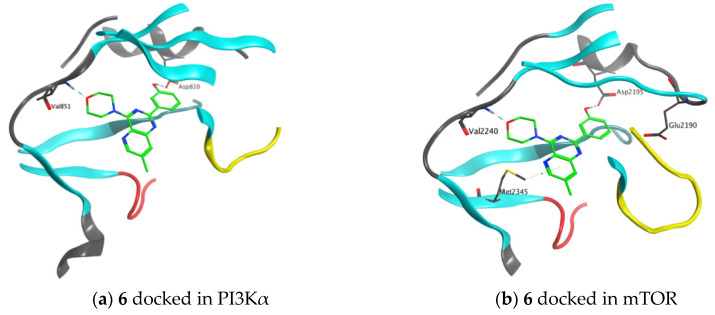
Binding modes for compound **6** on the two studied targets PI3Kα and mTOR.

In order to understand the duality observed with several derivatives, we focused on the docking solutions of derivative **6**. Compared to other compounds, the docking poses of **6** (Table 2, entries 2) specifically show an additional interaction in mTOR such as the π-CH interaction between the pyridine ring and the Met2345 residue (Figure 4).

**Figure 5:**
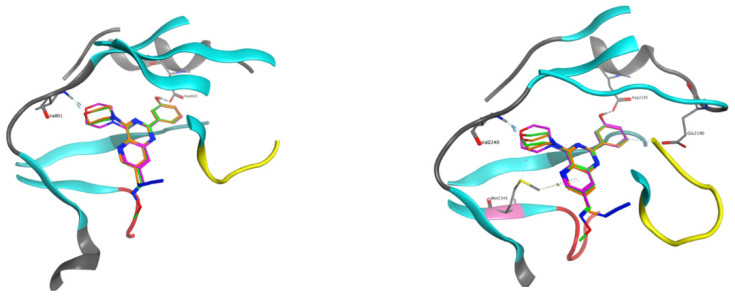
Binding modes from docking for compounds **6** (pink), **19** (orange) and **32** (green) in PI3Kα (**left**) and mTOR (**right**).

While compounds **6**, **19** and **32** present higher activity on PI3Kα versus mTOR, docking results show similar binding modes in both kinases (Figure 5). Additional computational experiments, such as molecular dynamics simulations or desolvation thermodynamics by displacing water molecules in the active site are needed to understand this discrepancy.

### 2.4. Cell Assays

The most active derivative **32** on PI3Kα was first tested on six different cell lines, i.e., hepatocellular carcinoma Huh-7, colorectal adenocarcinoma Caco-2, mammary carcinoma MDA-MB231, spontaneously immortalized keratinocytes Hacat, and normal human fibroblast (Table 3).

This compound exhibited the same activity profile and induced a cytotoxicity in the micromolar range for Huh-7, Caco-2, Hacat and normal human fibroblasts thereby attesting a good cellular penetration, while MDA-MB231 cells were less sensitive. This resistance might be due to the overactivation of the Ras/MAPK pathway in these cells which harbor activating mutation in Ras and Raf [42]. Indeed, PI3K inhibition might not compensate for this pathway overactivation.

**Figure 6:**
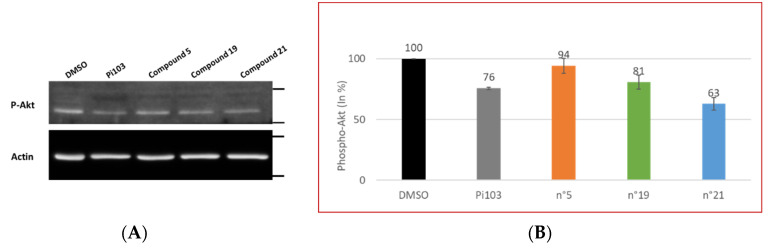
(**A**) Effects of Pi103 and compounds **5**, **19** and **21** on Akt phosphorylation (T308) in Caco-2 cells at 1 μM by Western blotting. (**B**) Quantification of three independent experiments. The values obtained for P-Akt were first normalized with actin and then normalized to 100%.

Compounds **5**, **19** and **21**, which inhibit the two kinase of interest, were then tested on the same cell lines panel. This time, fibroblasts were less sensitive than Huh-7, Caco-2 and Hacat cell lines. This result suggests that these compounds have more effects on fast proliferating cells such as cancer and immortalized cells lines with the exception of MDA-MB231 cells. Furthermore, on Caco-2 cells, compound **21** appeared to be the more cytotoxic while compound **19** appeared to have an intermediate effect between compounds **21** and **5**. To further understand the origins of these different cytotoxicities, Akt phosphorylation (on T308) was analyzed by Western blot after treatment of Caco-2 cells with **5**, **19** and **21** compounds. Figure 6A,B shows that compounds **5**, **19** and **21** differentially affect Akt phosphorylation in cells and that their degree of cytotoxicity on Caco-2 survival perfectly correlates with their ability to inhibit PI3K pathway. By analogy, the best cytotoxic values displayed by **21** on all the other cell lines, especially MDA-MB-231 might be explained by a stronger PI3K pathway inhibition—indeed **21** possesses an additional effect on the PI3Kβ isoform—rather than an impact of triazole on other cell target(s).

## 3. Materials and Methods

### 3.1. Chemistry

**General procedure A:** To a solution of halogenated derivative (1.0 eq.), in 1.2-dimethoxyethane was added boronic acid (1.5 eq.). An aqueous solution (1 M) of potassium carbonate (3.0 eq.) was then injected and the mixture was degassed by argon bubbling for 15 min. Pd(PPh_3_)_4_ (0.05 eq.) was added and the mixture was heated to 150 °C for 1 h by microwave irradiation. The solvent was removed in vacuo. The crude product was purified by flash chromatography.

**General procedure B:** A solution of halogenated derivative (1.0 eq.), potassium carbonate (3.0 eq.), 4.4.6-trimethyl-2-vinyl-1.3,2-*d*ioxaborinane (2.0 eq.), in dry toluene/ethanol, (3/1 mL) was degassed by argon bubbling for 15 min. Pd(PPh_3_)_4_ (0.05 eq.) was added and the mixture was heated to 150 °C for 1 h by microwave irradiation. The solvent was removed in vacuo. The crude product was purified by flash chromatography.

**General procedure C**: To a solution of MOM protected compound (1.0 eq.), in dioxane was added a solution of HCl in dioxane, 4.0 M (6.0 eq.). The mixture was stirred at room temperature until completion monitored by TLC. The solvent was removed by filtration and the product was washed with diethyl ether prior to drying the solid under reduced pressure.

**General procedure D:** To a solution of aldehyde in dry CH_2_Cl_2_/DMF, 6/1 (6 mL), was added the secondary amine. After cooling the reaction to 0 °C, NaBH(OAc)_3_ (5.0 eq.) was added. After 10 min of stirring, four drops of acetic acid were added to the mixture. The reaction was stirred at room temperature for 5 h before adding water (5 mL) and extracting the product. The combined organic layers were washed with a solution of saturated NaHCO_3_ (2 × 10 mL) and dried over MgSO_4_ and filtered. The solvent was removed under reduced pressure. The crude product then underwent the reaction as described in general procedure **C** to afford the final compound.

***2,4,7-Trichloropyrido[3,2-d]pyrimidine* (2)** [21]. To a solution of **1** [20] (1.0 g, 6.13 mmol) in phosphorus oxychloride (10 mL) was added phosphorus pentachloride (7.65 g, 36.78 mmol, 6.0 eq.). The mixture was heated by microwave irradiation at 160 °C for 2 h. The crude product was dissolved in CH_2_Cl_2_ (100 mL) and was then poured in ice. The mixture was stirred at room temperature for 6 h and then extracted. The combined organic layers were washed with water (2 × 20 mL) and dried over MgSO_4_ and filtered. The solvent was removed under reduced pressure. The crude product was purified by flash chromatography on silica gel (CH_2_Cl_2_/petroleum ether, 5/5) to afford **2** as a yellow solid (0.934 g, 65%). ^1^H NMR (400 MHz, CDCl_3_) δ: 8.31 (d, 1H, *J* = 2.2 Hz), 9.03 (d, 1H, *J* = 2.2 Hz). HRMS (EI-MS): *m/z* calculated for C_7_H_3_Cl_3_N_3_ [M + H]^+^: 234.9314; found 234.9323.

***2,7-Dichloro-4-morpholinylpyrido[3,2-d]pyrimidine* (3).** To a solution of **2** (0.755 g, 3.22 mmol) in THF (33 mL) were added successively morpholine (0.28 mL, 3.22 mmol, 1.0 eq.) and triethylamine (0.49 mL, 3.54 mmol, 1.1 eq.). The mixture was stirred at room temperature for 12 h and the solvent was removed under reduced pressure. The crude product was dissolved in CH_2_Cl_2_ (30 mL) and the organic layer was washed with a saturated solution of NaHCO_3_ (2 × 10 mL). The organic layer was dried over MgSO_4_ and filtered. The solvent was removed under reduced pressure. Purification by flash chromatography on silica gel (CH_2_Cl_2_/MeOH, 99/1) yielded **3** as a yellow solid (0.835 g, 91%). R_f_ (CH_2_Cl_2_/MeOH, 99/1): 0.11. Mp: 201–203 °C. IR (ATR diamond, cm^−1^) ν: 3043, 2966, 1546, 1411, 1334, 1254, 1108, 927, 865, 686. ^1^H NMR (250 MHz, CDCl_3_) δ: 3.87 (m, 4H, 2 × CH_2_(N)), 4.53 (m, 4H, 2 × CH_2_(O)), 7.99 (d, 1H, *J* = 2.5 Hz), 8.58 (d, 1H, *J* = 2.5 Hz). ^13^C NMR (62.5 MHz, CDCl_3_) δ: 47.8 (2 × CH_2_), 67.1 (2 × CH_2_), 130.7 (Cq), 133.7 (CH), 135.2 (Cq), 145.5 (CH), 149.4 (Cq), 159.2 (Cq), 168.2 (Cq) HRMS (EI-MS): *m/z* calculated for C_11_H_11_Cl_2_N_4_O [M + H]^+^: 286.0232; found 286.0302.

***7-Chloro-2-(3-methoxymethoxyphenyl)-4-morpholinylpyrido[3,2-d]pyrimidine* (4).** The reaction was carried out as described in general procedure **A** using **3** and 3-methoxymethoxyphenylboronic acid as the boronic acid. Purification by flash chromatography on silica gel (petroleum ether/EtOAc, 8/2) yielded **4** as a yellow solid (193 mg, 71%). R_f_ (petroleum ether/EtOAc, 8/2): 0.10. Mp: 196–198 °C. IR (ATR diamond, cm^−1^) ν: 2950, 1516, 1454, 1344, 1307, 1266, 1148, 1074, 1009, 874, 731. ^1^H NMR (250 MHz, CDCl_3_) δ: 3.53 (s, 3H, CH_3_), 3.92 (m, 4H, 2 × CH_2_(N)), 4.57 (m, 4H, 2 × CH_2_(O)), 5.28 (s, 2H, CH_2_), 7.17 (ddd, 1H, *J* = 1.1 Hz, *J* = 2.4 Hz, *J* = 8.1 Hz), 7.40 (t, 1H, *J* = 8.1 Hz), 8.14 (m, 3H), 8.59 (d, 1H, *J* = 2.4 Hz). ^13^C NMR (62.5 MHz, CDCl_3_) δ: 48.3 (2 × CH_2_), 56.3 (CH_3_), 66.6 (CH_2_), 67.5 (CH_2_), 94.8 (CH_2_), 116.6 (CH), 118.7 (CH), 120.2 (Cq), 122.4 (CH), 129.6 (CH), 131.3 (Cq), 135.0 (CH), 139.8 (Cq), 145.2 (CH), 149.1 (Cq), 157.6 (Cq), 159.2 (Cq), 160.9 (Cq). HRMS (EI/MS): *m/z* calculated for C_19_H_20_ClN_4_O_3_ [M + H]^+^: 387.1146; found 387.1151.

***7-Chloro-2-(3-hydroxyphenyl)-4-morpholinylpyrido[3,2-d]pyrimidine* (5).** The reaction was carried out as described in general procedure **A** using **3** (200 mg, 0.701 mmol) and 3-hydroxyphenylboronic acid as the boronic acid (116 mg, 0.842 mmol, 1.2 eq.). Purification by flash chromatography on silica gel (petroleum ether/EtOAc, 7/3) yielded **5** as a yellow solid (172 mg, 71%). R_f_ (petroleum ether/EtOAc, 7/3): 0.10. Mp: 230-232 °C. IR (ATR diamond, cm^−1^) ν: 3301, 2853, 1527, 1425, 1370, 1270, 1229, 1107, 1022, 948, 876, 737. ^1^H NMR (400 MHz, DMSO-*d*_6_) δ: 3.85 (m, 4H, 2 × CH_2_(N)), 4.50 (m, 4H, 2 × CH_2_(O)), 6.95 (d, 1H, *J* = 5.0 Hz), 7.32 (dd, 1H, *J* = 2.5 Hz, *J* = 5.0 Hz), 7.90 (d, 1H, *J* = 2.5 Hz), 7.91 (s, 1H), 8.29 (s, 1H), 8.75 (s, 1H), 9.61 (s, 1H, OH). ^13^C NMR (101 MHz, DMSO-*d*_6_) δ: 47.8 (2 × CH_2_), 66.8 (2 × CH_2_), 114.9 (CH), 117.8 (CH), 119.1 (CH), 129.3 (CH), 130.6 (Cq), 133.5 (Cq), 134.2 (CH), 138.6 (Cq), 144.9 (Cq), 148.2 (CH), 157.4 (Cq), 158.3 (Cq), 159.8 (Cq). HRMS (EI-MS): *m/z* calculated for C_17_H_16_ClN_4_O_2_ [M + H]^+^: 343.0884; found 343.0956.

***3-(7-Methyl-4-morpholinylpyrido[3,2-d]pyrimidin-2-yl)phenol* (6).** To a solution of compound **5** (100 mg, 0.32 mmol) in THF (3.5 mL) under argon were added trimethylaluminium (2M in toluene, 0.35 mL, 0.70 mmol, 2.2 eq.) and Pd(PPh_3_)_4_ (36 mg, 0.032 mmol, 0.1 eq.). The mixture was refluxed for 16h, and after cooling, the solvent was evaporated. The crude residue was purified by column chromatography on silica gel (gradient CH_2_Cl_2_ 100 to CH_2_Cl_2_/MeOH 99/1) to give the desired product **6** (65 mg, 70%) as a yellow solid. R_f_ (petroleum ether/EtOAc, 7/3): 0.10. Mp: 230–232 °C. IR (ATR diamond, cm^−1^) ν: 3301, 2853, 1527, 1425, 1370, 1270, 1229, 1107, 1022, 948, 876, 737. ^1^H NMR (400 MHz, CDCl_3_) δ 2.49 (d, *J* = 1.0 Hz, 3H, CH_3_), 3.85 (m, 4H, 2 × CH_2_(N)), 4.58 (m, 4H, 2 × CH_2_(O)), 6.94 (ddd, *J* = 8.1 Hz, *J* = 2.2 Hz, *J* = 1.0 Hz, 1H), 7.33 (t, *J* = 8.1 Hz, 1H), 7.94-8.04 (m, 4H), 8.51 (d, *J* = 2.2 Hz, 1H). ^13^C NMR (101 MHz, CDCl_3_) δ 18.9 (CH_3_), 48.2 (2 × CH_2_), 67.4 (2 × CH_2_), 115.5 (CH), 117.7 (CH), 121.1 (CH), 129.8 (CH), 131.1 (Cq), 135.1 (CH), 137.6 (Cq), 140.1 (Cq), 148.1 (CH+Cq), 156.2 (Cq), 159.4 (Cq), 160.1 (Cq). HRMS (EI-MS): *m/z* calculated for C_18_H_19_N_4_O_2_ [M + H]^+^: 323.1502; found 323.1503.

***2-(3-Methoxymethoxyphenyl)-4-morpholinyl-7-vinylpyrido[3,2-d]pyrimidine* (7).** The reaction was carried out as described in general procedure **B** using **4** to afford **7** as a yellow solid (178 mg, 91%). R_f_ (petroleum ether/EtOAc, 8/2): 0.16. Mp: 105–107 °C. IR (ATR diamond, cm^−1^) ν: 2856, 1527, 1487, 1454, 1343, 1275, 1111, 1070, 1021, 956, 910, 739. ^1^H NMR (400 MHz, CDCl_3_) δ: 3.52 (s, 3H, CH_3_), 3.96–3.89 (m, 4H, 2 × CH_2_(N)), 4.58 (m, 4H, 2 × CH_2_(O)), 5.28 (s, 2H, CH_2_), 5.56 (d, 1H, *J* = 11.0 Hz, CH_2alkene_), 6.05 (d, 1H, *J* = 17.7 Hz, CH_2alkene_), 6.84 (dd, 1H, *J* = 11.0 Hz, *J* = 17.7 Hz, CH_alkene_), 7.20–7.13 (m, 1H), 7.40 (t, 1H, *J* = 7.9 Hz), 8.19–8.09 (m, 3H), 8.72 (d, 1H, *J* = 1.9 Hz). ^13^C NMR (101 MHz, CDCl_3_) δ: 48.3 (2 × CH_2_), 56.3 (CH_3_), 67.5 (2 × CH_2_), 94.7 (CH_2_), 116.5 (CH), 118.4 (CH), 119.0 (CH_2_), 122.3 (CH), 129.5 (CH), 132.6 (CH), 133.2 (CH), 136.2 (Cq), 139.8 (Cq), 140.2 (Cq), 144.9 (CH), 148.4 (Cq), 157.6 (Cq), 159.4 (Cq), 160.2 (Cq). HRMS (EI/MS): *m/z* calculated for C_21_H_23_ClN_4_O_3_ [M + H]^+^: 379.1765; found 379.1766.

***7-(3-(Methoxymethoxy)phenyl)-4-morpholinylpyrido[3,2-d]pyrimidine-7-carbaldehyde* (8).** To a solution of **7** (400 mg, 1.06 mmol) in THF/H_2_O, 1/1 (30 mL) was added osmium tetroxide, 2.5% wt (0.76 mL, 0.053 mmol, 0.05 eq.). The mixture was stirred until it turned dark, then NaIO_4_ (680 mg, 3.18 mmol, 3.0 eq.) was added was added in three equal portions. The solution was stirred during 2 h at room temperature followed by the addition of an aqueous solution of Na_2_S_2_O_3_ 10% wt. After filtration on celite, the filtrate was diluted in EtOAc (50 mL), the combined organic layers were washed with water (2 × 20 mL) and dried over MgSO_4_ and filtered. The solvent was removed under reduced pressure. The crude product was purified by flash chromatography on silica gel (petroleum ether/EtOAc, 8/2) to afford **8** as a yellow solid (395 mg, 98%). R_f_ (petroleum ether/EtOAc, 8/2): 0.20. Mp: 141–143 °C. IR (ATR diamond, cm^−1^) ν: 2911, 1701, 1508, 1461, 1426, 1268, 1154, 1116, 1071, 1008, 957, 739. ^1^H NMR (400 MHz, DMSO-*d*_6_) δ: 3.43 (s, 3H, CH_3_), 3.83 (m, 4H, 2 × CH_2_(N)), 4.52 (m, 4H, 2 × CH_2_(O)), 5.28 (s, 2H, CH_2_), 7.25–7.13 (m, 1H), 7.45 (t, 1H, *J* = 7.5 Hz), 8.10 (d, 2H, *J* = 7.4 Hz), 8.67 (s, 1H,), 9.09 (s, 1H), 10.28 (s, 1H, CHO). ^13^C NMR (101 MHz, DMSO-*d*_6_) δ: 47.8 (2 × CH_2_), 55.6 (CH_3_), 66.3 (2 × CH_2_), 94.0 (CH_2_), 115.7 (CH), 118.5 (CH), 121.7 (CH), 129.6 (CH), 133.3 (Cq), 135.1 (Cq), 138.9 (CH), 139.2 (Cq), 144.4 (CH), 147.4 (Cq), 157.0 (Cq), 158.5 (Cq), 159.7 (Cq), 192.4 (CH). HRMS (EI/MS): *m/z* calculated for C_20_H_21_ClN_4_O_4_ [M + H]^+^: 381.1557; found 381.1560.

***2-(3-Hydroxyphenyl)-4-morpholinyl-7-vinylpyrido[3,2-d]pyrimidine* (9).** The reaction was carried out as described in general procedure **C** using **7** (150 mg, 0.396 mmol) to afford **9** as a white solid (130 mg, 98%). R_f_ (petroleum ether/EtOAc, 8/2): 0.08. Mp: 183–185 °C. IR (ATR diamond, cm^−1^) ν: 3338, 2856, 1597, 1531, 1483, 1438, 1230, 1107, 968, 858, 739. ^1^H NMR (400 MHz, CDCl_3_) δ: 3.89 (m, 4H, 2 × CH_2_(N)), 4.54 (m, 4H, 2 × CH_2_(O)), 5.51 (d, 1H, *J* = 11.0 Hz, CH_2alkene_), 5.99 (d, 1H, *J* = 17.6 Hz, CH_2alkene_), 6.77 (dd, 1H, *J* = 11.0 Hz, *J* = 17.6 Hz, CH_alkene_), 6.92 (d, 1H, *J* = 7.5 Hz), 7.29 (t, 1H, *J* = 7.5 Hz), 7.98 (s, 2H), 8.08 (m, 2H), 8.70 (d, 1H, *J* = 7.5 Hz). ^13^C NMR (101 MHz, CDCl_3_) δ: 48.3 (2 × CH_2_), 66.1 (CH_2_), 67.5 (CH_2_), 115.6 (CH), 118.0 (CH), 119.2 (CH_2_), 121.0 (CH), 129.8 (CH), 132.2 (CH), 132.3 (Cq), 133.1 (CH), 136.4 (Cq), 140.0 (Cq), 144.9 (CH), 148.2 (Cq), 156.4 (Cq), 159.3 (Cq), 160.5 (Cq). HRMS (EI/MS): *m/z* calculated for C_19_H_19_N_4_O_2_ [M + H]^+^: 335.1430; found 335.1504.

***2-(3-Hydroxyphenyl)-4-morpholinylpyrido[3,2-d]pyrimidine-7-carbaldehyde* (10).** The reaction was carried out as described in general procedure **G** using **8** (200 mg, 0.526 mmol) to afford **10** as a yellow solid (159 mg, 90%). R_f_ (petroleum ether/EtOAc, 8/2): 0.05. Mp: 182–184 °C. IR (ATR diamond, cm^−1^) ν: 2852, 1695, 1556, 1516, 1426, 1377, 1283, 1107, 1025, 882, 743. ^1^H NMR (400 MHz, DMSO-*d*_6_) δ: 3.84 (m, 4H, 2 × CH_2_(N)), 4.52 (bs, 4H, 2 × CH_2_(O)), 6.92 (m, 1H), 7.30 (t, 1H, *J* = 8.1 Hz), 7.90 (m, 2H), 8.64 (d, 1H, *J* = 1.9 Hz), 9.09 (d, 1H, *J* = 1.9 Hz), 9.58 (s, 1H, OH), 10.28 (s, 1H, CHO). ^13^C NMR (101 MHz, DMSO-*d*_6_) δ: 48.1 (2 × CH_2_), 66.3 (2 × CH_2_), 114.9 (CH), 117.8 (CH), 119.1 (CH), 129.4 (CH), 133.3 (Cq), 136.1 (Cq), 138.8 (CH), 139.0 (Cq), 144.3 (CH), 147.4 (Cq), 157.5 (Cq), 158.4 (Cq), 159.7 (Cq), 192.4 (CH). HRMS (EI/MS) *m/z* calculated for C_18_H_17_N_4_O_3_ [M + H]^+^: 337.1563; found 337.1546.

***3-(7-((Cyclopropylamino)methyl)-4-morpholinylpyrido[3,2-d]pyrimidin-2-yl)phenol hydrochloride salt* (11).** To a solution of **8** (70 mg, 0.184 mmol) in dry CH_2_Cl_2_ (6 mL), was added a small amount of MgSO_4_ and cyclopropylamine (12 µL, 0.184 mmol, 1.0 eq.). The reaction was stirred at room temperature for 12 h before filtering the MgSO_4_ and removing the solvent in vacuo. The crude product was diluted in methanol (6 mL) and NaBH_3_CN (60 mg, 0.92 mmol, 5.0 eq.) was added to the mixture. Once the reaction stopped bubbling (15 min), the crude product was extracted. The combined organic layers were washed with a solution of saturated NaHCO_3_ (2 × 10 mL) and dried over MgSO_4_ and filtered. The crude product was then subjected to the general procedure **C** to afford **11** as a white solid (28 mg, 40%). R_f_ (CH_2_Cl_2_/MeOH, 99/1): 0.05. Mp: 244–246 °C. IR (ATR diamond, cm^−1^) ν: 3351, 3047, 1613, 1552, 1517, 1428, 1385, 1310, 1114, 1024, 864, 732. ^1^H NMR (400 MHz, DMSO-*d*_6_) δ: 0.81 (s, 2H, 2xH_cypro_), 1.09 (s, 2H 2×H_cypro_), 2.72 (s, 1H, H_cypro_), 3.87 (m, 4H, 2 × CH_2_(N)), 4.48 (s, 2H, CH_2_), 4.64 (m, 4H, 2 × CH_2_(O)), 7.06 (s, 1H), 7.38 (s, 1H), 7.91 (d, 2H, *J* = 11.7 Hz), 8.70 (s, 1H), 9.10 (s, 1H), 9.38 (s, 1H, OH), 10.39 (s, 1H, NH). ^13^C DEPT NMR (101 MHz, DMSO-*d*_6_) δ 10.2 (2 × CH_2_), 28.3 (CH), 48.9 (2 × CH_2_), 51.6 (CH_2_), 66.7 (2 × CH_2_), 112.9 (CH), 116.9 (CH), 120.1 (CH), 131.6 (CH), 134.9 (CH), 150.1 (CH). HRMS (EI/MS): *m/z* calculated for C_21_H_24_N_5_O_2_ [M + H]^+^: 378.1852; found 378.1927.

***3-(4-Morpholinyl-7-(morpholinylmethyl)pyrido[3,2-d]pyrimidin-2-yl)phenol hydrochloride salt* (12).** The reaction was carried out as described in general procedure **D** using **8** (80 mg, 0.21 mmol) and morpholine to afford **12** as a white solid (52 mg, 61%) R_f_ (CH_2_Cl_2_/MeOH, 99/1): 0.04. Mp: 239–141 °C. IR (ATR diamond, cm^−1^) ν: 3344, 3037, 1552, 1510, 1417, 1292, 1114, 1028, 864, 736. ^1^H NMR (400 MHz, DMSO-*d*_6_) δ: 3.26 (m, 4H, 2 × CH_2_(N)), 3.86 (m, 8H, 2 × CH_2_(N) and 2 × CH_2_(O)), 4.63 (m, 6H, CH_2_ and 2 × CH_2_(O)), 7.03 (s, 1H), 7.37 (s, 1H), 7.96 (m, 2H), 8.76 (s, 1H), 9.13 (s, 1H, H_6_), 12.35 (s, 1H, NH). ^13^C DEPT NMR (101 MHz, DMSO-*d*_6_) δ: 18.4 (2CH_2_), 54.4 (2CH_2_), 59.1 (2CH_2_), 66.4 (2CH_2_), 66.8 (2CH_2_), 114.1 (CH), 117.9 (CH), 119.9 (CH), 130.4 (CH), 136.2 (CH), 147.1 (CH) HRMS (EI/MS): *m/z* calculated for C_22_H_26_N_5_O_3_ [M + H]^+^: 408.1957; found 408.2020

***2-(3-Hydroxyphenyl)-7-(4-methylpiperazin-1-ylmethyl)-4-morpholinylpyrido[3,2-d]pyrimidine hydrochloride salt* (13).** The reaction was carried out as described in general procedure **D** using **8** (100 mg, 0.21 mmol,) and *N*-methylpiperazine to afford **13** as a white solid (120 mg, 92%). R_f_ (CH_2_Cl_2_/MeOH, 99/1): 0.04. Mp: 243–245 °C. IR (ATR diamond, cm^−1^) ν: 2927, 1616, 1556, 1508, 1420, 1388, 1312, 1112, 881, 729. ^1^H NMR (400 MHz, DMSO-*d*_6_) δ: 2.81 (s, 3H, NCH_3_), 3.46 (m, 2H, CH_2_(N)), 3.88 (s, 5H), 4.86–4.45 (m, 6H, 3 × CH_2_(N)), 7.10 (d, 1H, *J* = 7.2 Hz), 7.40 (t, 1H, *J* = 8.2 Hz), 7.99–7.76 (m, 2H), 8.81 (s, 1H), 9.10 (s, 1H, H_6_), 9.76 (bs, 1H, OH), 11.91 (s, 1H, NH). ^13^C DEPT NMR (101 MHz, DMSO-*d*_6_) δ: 48.4 (2 × CH_2_), 50.1 (CH_3_), 54.7 (2 × CH_2_), 57.8 (2 × CH_2_), 60.1 (CH_2_), 66.4 (2 × CH_2_), 115.6 (CH), 119.7 (2 × CH), 129.9 (CH), 145.4 (CH), 146.5 (CH), 148.5 (CH). HRMS (EI/MS): *m/z* calculated for C_23_H_28_N_6_O_2_ [M + H]^+^: 421.2274; found 421.2361.

***(2-(3-(Methoxymethoxy)phenyl)-4-morpholinylpyrido[3,2-d]pyrimidin-7-yl)methanol* (14).** To a solution of NaBH_4_ (60 mg, 1.58 mmol, 2.0 eq.) in MeOH (12 mL), was added **8** (300 mg, 0.79 mmol). The mixture was stirred at room temperature for two hours. The solvent was then removed under reduced pressure, the crude product was diluted in CH_2_Cl_2_ (30 mL), the organic layer was washed with brine (10 mL), dried over MgSO_4_, filtered and the solvent removed under reduced pressure to afford **14** as a yellow solid (298 mg, 99%). Rf (petroleum ether/EtOAc, 7/3): 0.1. Mp: 134–136 °C. IR (ATR diamond, cm^−1^) ν: 3265, 2913, 1532, 1491, 1438, 1356, 1262, 1152, 1115, 1066, 1009, 915, 874, 739. ^1^H NMR (400 MHz, DMSO-*d*_6_) δ: 3.54 (s, 3H, CH_3_), 3.90 (m, 4H, 2 × CH_2_(N)), 4.55 (m, 4H, 2 × CH_2_(O)), 4.82 (s, 2H, CH_2_OH), 5.29 (s, 2H, CH_2_), 7.18 (dd, 1H, *J* = 2.3 Hz, *J* = 8.1 Hz), 7.41 (t, 1H, *J* = 7.9 Hz), 8.12 (m, 3H), 8.55 (d, 1H, *J* = 1.7 Hz), 9.54 (bs, 1H, OH). ^13^C NMR (101 MHz, DMSO-*d*_6_) δ: 48.3 (2 × CH_2_), 56.3 (CH3), 62.3 (2 × CH_2_), 67.4 (CH2), 94.7 (CH2), 116.5 (CH), 118.5 (CH), 122.3 (CH), 129.6 (CH), 132.2 (Cq), 133.4 (CH), 139.9 (Cq), 140.5 (Cq), 145.5 (CH), 148.0 (Cq), 157.6 (Cq), 159.3 (Cq), 160.0 (Cq). HRMS (EI/MS): *m/z* calculated for C_20_H_23_N_4_O_4_ [M + H]^+^: 383.1641; found 383.1715.

***3-(7-(Hydroxymethyl)-4-morpholinylpyrido[3,2-d]pyrimidin-2-yl)phenol* (15).** The reaction was carried out as described in general procedure **C** using **14** (200 mg, 0.523 mmol) to afford **15** as a yellow solid (154 mg, 87%). R_f_ (CH_2_Cl_2_/MeOH, 97/3): 0.20. Mp: 229–231 °C. IR (ATR diamond, cm^−1^) ν: 3248, 2851, 1511, 1458, 1356, 1238, 1111, 1054, 886, 735. ^1^H NMR (400 MHz, DMSO-*d*_6_) δ: 3.82 (m, 4H, 2 × CH_2_(N)), 4.51 (m, 4H, 2 × CH_2_(O)), 4.73 (d, 2H, *J* = 5.6 Hz, CH_2_OH), 5.59 (t, 1H, OH), 6.89 (dd, 1H, *J* = 1.6 Hz, *J* = 7.9 Hz), 7.29 (t, 1H, *J* = 7.9 Hz), 7.89 (m, 2H), 8.04 (s, 1H), 8.70 (s, 1H), 9.54 (s, 1H, OH). ^13^C NMR (101 MHz, DMSO-*d*_6_) δ: 47.6 (2 × CH_2_), 60.3 (CH_2_), 66.4 (2 × CH_2_), 114.8 (CH), 117.5 (CH), 119.0 (CH), 129.3 (CH), 131.1 (Cq), 132.5 (CH), 139.3 (Cq), 142.2 (Cq), 145.8 (CH), 147.6 (Cq), 157.4 (Cq), 158.5 (Cq), 158.8 (Cq). HRMS (EI/MS): *m/z* calculated for C_18_H_19_N_4_O_3_ [M + H]^+^: 339.1379; found 339.1441.

***3-(7-(Methoxymethyl)-4-morpholinylpyrido[3,2-d]pyrimidin-2-yl)methanol* (16).** To a solution of **8** (80 mg, 0.209 mmol) in THF (10 mL) at 0 °C, was added NaBH_4_ (8 mg, 0.23 mmol, 1.1 eq.) followed by the addition of MeI (13 µL, 0.209 mmol, 1.0 eq.). The solution was stirred at room temperature for 2 h 30 min before removing the solvent in vacuo. The crude product was diluted in CH_2_Cl_2_ (30 mL) and the organic layer was washed with brine (10 mL), dried over MgSO_4_, filtered and the solvent removed under reduced pressure. The crude product directly underwent the reaction described in general procedure **C** to afford **16** as a yellow solid (54 mg, 74%). R_f_ (CH_2_Cl_2_/MeOH, 99/1): 0.13. Mp: 165–167 °C. IR (ATR diamond, cm^−1^) ν: 3273, 2852, 1540, 1491, 1438, 1352, 1270, 1103, 1021, 968, 878, 739, 678. ^1^H NMR (400 MHz, CDCl_3_) δ: 3.46 (s, 3H, CH_3_), 3.92 (m, 4H, 2 × CH_2_(N)), 4.58 (m, 4H, 2 × CH_2_(O)), 4.63 (s, 2H, CH_2_), 6.94 (dd, 1H, *J* = 2.2 Hz, *J* = 7.7 Hz), 7.38 (t, 1H, *J* = 7.9 Hz), 7.98 (m, 1H), 8.02 (d, 1H, *J* = 7.9 Hz), 8.10 (m, 1H), 8.65 (d, 1H, *J* = 2.1 Hz), 9.62 (s, 1H, OH). ^13^C NMR (101 MHz, CDCl_3_) δ: 48.3 (2 × CH_2_), 58.9 (CH_3_), 67.5 (2 × CH_2_), 72.0 (CH_2_), 115.5 (CH), 117.9 (CH), 121.1 (CH), 129.8 (CH), 132.5 (Cq), 134.5 (CH), 137.7 (Cq), 140.1 (Cq), 146.1 (CH), 148.1 (Cq), 156.2 (Cq), 159.4 (Cq), 160.3 (Cq). HRMS (EI/MS): *m/z* calculated for C_19_H_21_N_4_O_3_ [M + H]^+^: 353.1535; found 353.1609.

***4-(7-(Iodomethyl)-2-(3-(methoxymethoxy)phenyl)pyrido[3,2-d]pyrimidin-4-yl)morpholine* (17).** To a solution of triphenylphosphine (137 mg, 0.523 mmol, 2.0 eq.) in CH_2_Cl_2_ (5 mL) at 0 °C, was added iodine (199 mg, 0.784 mmol, 3.0 eq.) and imidazole (36 mg, 0.523 mmol, 2.0 eq.) and then **8** (100 mg, 0.262 mmol). The reaction was stirred at 0 °C for 7 h before adding an aqueous solution of Na_2_S_2_O_3_ 10% (5 mL). After 15 min of stirring, the mixture was extracted and the organic layer was washed with water (5 mL), dried over MgSO_4_, filtered, and the solvent removed under reduced pressure. The crude product was purified by flash chromatography on silica gel (petroleum ether/EtOAc, 1/9) to afford **17** as a yellow solid (89 mg, 69%). R_f_ (petroleum ether/EtOAc, 1/9): 0.14. Mp: 144–146 °C. IR (ATR diamond, cm^−1^) ν: 2921, 1732, 1663, 1532, 1491, 1430, 1270, 1234, 1148, 1107, 1021, 964, 874, 739. ^1^H NMR (400 MHz, CDCl_3_) δ: 3.53 (s, 3H, CH_3_), 3.92 (m, 4H, 2 × CH_2_(N)), 4.53 (s, 2H, CH_2_), 4.58 (m, 4H, 2 × CH_2_(O)), 5.28 (s, 2H, CH_2_), 7.17 (m, 1H), 7.40 (t, 1H, *J* = 7.9 Hz), 8.17–8.10 (m, 3H), 8.66 (d, 1H, *J* = 2.3 Hz). ^13^C NMR (101 MHz, CDCl_3_) δ: 48.3 (2 × CH_2_), 56.3 (CH_3_), 62.4 (CH_2_), 67.4 (2 × CH_2_), 94.8 (CH_2_), 116.5 (CH), 118.5 (CH), 122.4 (CH), 129.6 (CH), 132.2 (Cq), 133.4 (CH), 135.4 (Cq), 140.0 (Cq), 145.5 (CH), 148.0 (Cq), 157.6 (Cq), 159.3 (Cq), 160.0 (Cq). HRMS (EI/MS): *m/z* calculated for C_20_H_22_IN_4_O_3_ [M + H]^+^: 493.0658; found 493.0738.

***4-(7-(Azidomethyl)-2-(3-(methoxymethoxy)phenyl)pyrido[3,2-d]pyrimidin-4-yl) morpholine* (18).** To a solution of **17** (78 mg, 0.158 mmol) in DMF (5 mL) was added sodium azide (15 mg, 0.238 mmol, 1.5 eq.). The reaction was stirred at 65 °C for 6 h, before diluting the solution in CH_2_Cl_2_ (60 mL). The combined organic layers were washed with an aqueous solution of citric acid, 10% (10 mL), a saturated solution of NaHCO_3_ (10 mL) and then were washed twice with water (10 mL), dried over MgSO_4_, filtered and the solvent removed under reduced pressure. The crude product was purified by flash chromatography on silica gel (CH_2_Cl_2_/MeOH, 98/2) to afford **18** as a yellow solid (49 mg, 75%). R_f_ (CH_2_Cl_2_/MeOH, 98/2): 0.11. Mp: 143–145 °C. IR (ATR diamond, cm^−1^) ν: 2872, 2086, 1612, 1523, 1428, 1307, 1109, 1021, 862, 731. ^1^H NMR (400 MHz, CDCl_3_) δ: 3.53 (s, 3H, CH_3_), 3.88 (m, 4H, 2 × CH_2_(N)), 4.68 (m, 4H, 2 × CH_2_(O)), 4.90 (s, 2H, CH_2_N_3_), 5.28 (s, 2H, CH_2_), 7.08 (m, 1H), 7.41 (m, 1H), 7.87 (m, 2H), 8.46 (s, 1H), 8.91 (s, 1H). ^13^C NMR (101 MHz, CDCl_3_) δ: 48.4 (2 × CH_2_), 50.4 (CH_2_), 56.3 (CH_3_), 66.3 (2 × CH_2_), 94.7 (CH_2_), 115.5 (CH), 119.6 (2 × CH), 130.0 (CH), 132.2 (Cq), 134.8 (Cq), 137.8 (CH), 140.2 (Cq), 147.5 (CH), 149.2 (Cq), 157.8 (Cq), 159.5 (Cq), 160.3 (Cq). HRMS (EI/MS): *m/z* calculated for C_20_H_22_N_7_O_3_ [M + H]^+^: 408.1706; found 408.1803.

***3-(7-(Azidomethyl)-4-morpholinylpyrido[3,2-d]pyrimidin-2-yl)phenol* (19).** The reaction was carried out as described in general procedure **C** using **18** (40 mg, 0.10 mmol) to afford **19** as a yellow solid (35 mg, 98%). R_f_ (CH_2_Cl_2_/MeOH, 99/1): 0.05. Mp: 169–171 °C. IR (ATR diamond, cm^−1^) ν: 3383, 3043, 2868, 2096, 1613, 1552, 1511, 1434, 1307, 1111, 1021, 862, 731, 670. ^1^H NMR (400 MHz, CDCl_3_) δ: 3.89 (m, 4H, 2 × CH_2_(O)), 4.70 (bs, 4H, 2 × CH_2_(N)), 4.89 (s, 2H, CH_2_N_3_), 7.12 (m, 1H), 7.43 (m, 1H), 7.87 (m, 2H), 8.49 (s, 1H), 8.87 (s, 1H), 10.06 (bs, 1H, OH). ^13^C NMR (101 MHz, CDCl_3_) δ: 48.4 (2 × CH_2_), 50.4 (CH_2_), 66.3 (2 × CH_2_), 115.4 (CH), 119.6 (2 × CH), 130.0 (CH), 132.7 (Cq), 133.8 (Cq), 137.4 (CH), 139.9 (Cq), 146.9 (CH), 148.8 (Cq), 157.8 (Cq), 159.1 (Cq), 160.0 (Cq). HRMS (EI/MS): *m/z* calculated for C_18_H_18_N_7_O_2_ [M + H]^+^: 364.1444; found 364.1517.

***3-(7-((4-((Dimethylamino)methyl)-1H-1,2,3-triazol-1-yl)methyl)-4-morpholinylpyrido[3,2-d]pyrimidin-2-yl)phenol* (20).** To a solution of **19** (80 mg, 0.22 mmol) in MeCN (3 mL) were added CuOAc.H_2_O (25 µL, C = 0.4 M, 0.011 mmol, 0.05 eq.), 3-dimethylamino-1-propyne (26 µL, 0.22 mmol, 1.0 eq.) and a few drops of triethylamine until the products were soluble in the solvent. The reaction was stirred at room temperature for 12 h, before removing the solvent in vacuo. The crude product was dissolved in CH_2_Cl_2_ (30 mL) and the organic layer was washed with a saturated solution of NaHCO_3_ (10 mL) and brine (10 mL). The organic layer was dried over MgSO_4_ and filtered. The solvent was removed under reduced pressure. The crude product was purified by precipitation with CH_2_Cl_2_ to afford **20** as a yellow solid (30 mg, 31%). Mp: 224–226 °C. IR (Diamond ATR, cm^−1^) ν: 3271, 2856, 1595, 1557, 1508, 1437, 1308, 1269, 1166, 1113, 1062, 1029, 968, 792, 739, 674, ^1^H NMR (400 MHz, DMSO-*d*_6_) δ: 2.18 (bs, 6H, 2 × CH_3_), 3.55 (bs, 2H, CH_2_), 3.83 (m, 4H, 2 × CH_2_(N)), 4.51 (m, 4H, 2 × CH_2_(O)), 5.89 (s, 2H, CH_2_OCH_3_), 6.90 (d, 1H, *J* = 7.8 Hz), 7.30 (t, 1H, *J* = 8.0 Hz), 7.87 (d, 2H, *J* = 6.6 Hz), 7.94 (s, 1H), 8.24 (s, 1H, CH), 8.73 (s, 1H, H_6_), 9.55 (s, 1H, OH). ^13^C NMR (101 MHz, DMSO-*d*_6_) δ: 45.0 (2 × CH_3_), 48.1 (2 × CH_2_), 50.4 (CH_2_), 55.4 (CH_2_), 66.8 (2 × CH_2_), 115.3 (CH), 118.1 (CH), 119.5 (CH), 124.8 (CH), 129.8 (CH), 132.3 (Cq), 135.0 (CH), 136.3 (Cq), 139.5 (Cq), 144.4 (Cq), 146.4 (CH), 147.8 (Cq), 157.9 (Cq), 158.8 (Cq), 159.6 (Cq). HRMS (EI-MS): *m/z* calculated for C_23_H_27_N_8_O_2_ [M + H]^+^, 447.2257 found 447.2251.

***3-(7-((4-(Methoxymethyl)-1H-1.2.3-triazol-1-yl)methyl)-4-morpholinylpyrido[3,2-d]pyrimidin-2-yl)phenol* (21).** To a solution of **19** (80 mg, 0.22 mmol, 1.0) in MeCN (4 mL) were added CuI (3 mg, 0.01 mmol, 0.05 eq.), 3-methoxy-1-propyne (19 µL, 0.22 mmol, 1.0 eq.) and a few drops of triethylamine until the products were soluble in the solvent. The reaction was stirred at room temperature for 12 h, before removing the solvent in vacuo. The crude product was dissolved in EtOAc (30 mL) and the organic layer was washed with a saturated solution of NaHCO_3_ (10 mL) and brine (10 mL). The organic layer was dried over MgSO_4_ and filtered. The solvent was removed under reduced pressure. The crude product was purified by flash chromatography on silica gel (CH_2_Cl_2_/MeOH, 99/1) to afford **21** as a yellow solid (73 mg, 79%). R_f_ (CH_2_Cl_2_/MeOH, 99/1): 0.05. Mp: 241–243 °C. IR (ATR diamond, cm^−1^) ν: 3130, 2856, 1589, 1552, 1516, 1430, 1315, 1275, 1107, 1062, 1029, 968, 792, 739, 674. ^1^H NMR (400 MHz, DMSO-*d*_6_) δ: 3.28 (s, 3H, CH_3_), 3.81 (m, 4H, 2 × CH_2_(N)), 4.47 (m, 6H, CH_2_ and 2 × CH_2_(O)), 5.88 (s, 2H, CH_2_(O)), 6.89 (d, 1H, *J* = 7.8 Hz), 7.28 (t, 1H, *J* = 8.0 Hz), 7.87 (d, 2H, *J* = 6.6 Hz), 7.94 (s, 1H), 8.32 (s, 1H, CH), 8.72 (s, 1H), 9.53 (s, 1H, OH). ^13^C NMR (101 MHz, DMSO-*d*_6_) δ: 47.6 (2 × CH_2_), 49.9 (CH_2_), 57.4 (CH_3_), 64.9 (CH_2_), 66.4 (2 × CH_2_), 114.8 (CH), 117.6 (CH), 119.0 (CH), 124.5 (CH), 129.3 (CH), 131.8 (Cq), 134.5 (CH), 135.8 (Cq), 139.0 (Cq), 144.4 (Cq), 145.9 (CH), 147.3 (Cq), 157.4 (Cq), 158.4 (Cq), 159.2 (Cq). HRMS (EI/MS): *m/z* calculated for C_22_H_24_N_7_O_3_ [M + H]^+^: 434.1862; found 434.1939.

***3-(7-((4-((Methoxymethoxy)methyl)-1H-1,2,3-triazol-1-yl)methyl)-4-morpholinyl pyrido[3,2-d]pyrimidin-2-yl)phenol* (22).** To a solution of **19** (45 mg, 0.13 mmol) in MeCN (4 mL) were added CuI (1.2 mg, 0.006 mmol, 0.05 eq.), methoxy(prop-2-yn-1-yloxy)methane (14 mg, 0.14 mmol, 1.1 eq.) and a few drops of triethylamine until the products were soluble in the solvent. The reaction was stirred at room temperature for 12 h, before removing the solvent in vacuo. The crude product was dissolved in CH_2_Cl_2_ (30 mL) and the organic layer was washed with a saturated solution of NaHCO_3_ (10 mL) and brine (10 mL). The organic layer was dried over MgSO_4_ and filtered. The solvent was removed under reduced pressure. The crude product was purified by flash chromatography on silica gel (CH_2_Cl_2_/MeOH, 98/2) to afford **22** as a brown pale solid (15 mg, 25%). Mp: 218–220 °C. IR (Diamond ATR, cm^−1^) ν: 3204, 2937, 1618, 1589, 1552, 1516, 1430, 1315, 1275, 1107, 1062, 1029, 968, 792, 739, 674. ^1^H NMR (400 MHz, CDCl_3_) δ: 3.29 (s, 3H, CH_3_), 3.82 (m, 4H, 2 × CH_2_(N)), 4.47 (m, 4H, 2 × CH_2_(O)), 4.61 (s, 2H, CH_2_), 4.65 (s. 2H, CH_2_), 5.90 (s, 2H, CH_2_), 6.89 (d, 1H, *J* = 7.8 Hz), 7.30 (t, 1H, *J* = 8.0 Hz), 7.88 (d, 2H, *J* = 6.6 Hz), 7.96 (s, 1H), 8.35 (s, 1H, CH), 8.74 (s, 1H), 9.55 (s, 1H, OH). ^13^C NMR (101 MH_z_, CDCl_3_) δ: 47.6 (2 × CH_2_), 49.9 (CH_2_), 56.6 (CH_3_), 61.7 (CH_2_), 68.2 (2 × CH_2_), 95.5 (CH_2_), 115.4 (CH), 118.0 (CH), 119.7 (CH), 122.9 (CH), 125.3 (CH), 129.8 (CH), 135.0 (Cq), 136.0 (Cq), 139.1 (Cq), 144.8 (Cq), 146.4 (CH), 147.8 (Cq), 157.9 (Cq), 158.8 (Cq), 159.5 (Cq). HRMS (EI-MS): *m/z* calculated for C_23_H_26_N_7_O_4_ [M + H]^+^, 464.2046 found 464.2041.

***3-(7-((4-(Hydroxymethyl)-1H-1,2,3-triazol-1-yl)methyl)-4-morpholinylpyrido[3,2-d]pyrimidin-2-yl)phenol* (23).** To a solution of **19** (82 mg, 0.23 mmol) in MeCN (4 mL) were added CuI (4 mg, 0.01 mmol, 0.05 eq.), 2-propyn-1-ol (16 µL, 0.25 mmol, 1.1 eq.) and a few drops of triethylamine until the products were soluble in the solvent. The reaction was stirred at room temperature for 12 h, before removing the solvent in vacuo. The crude product was dissolved in CH_2_Cl_2_ (30 mL) and the organic layer was washed with a saturated solution of NaHCO_3_ (10 mL) and brine (10 mL). The organic layer was dried over MgSO_4_ and filtered. The solvent was removed under reduced pressure. The crude product was purified by flash chromatography on silica gel (CH_2_Cl_2_/MeOH, 98/2) to afford **23** as a yellow solid (49 mg, 51%). R_f_ (CH_2_Cl_2_/MeOH, 98/2): 0.04. Mp: 238–240 °C. IR (ATR diamond, cm^−1^) ν: 3154, 2962, 2848, 1605, 1556, 1495, 1458, 1348, 1266, 1115, 1025, 886, 739, 678. ^1^H NMR (400 MHz, DMSO-*d*_6_) δ: 3.81 (m, 4H, 2 × CH_2_(N)), 4.50 (s, 4H, 2 × CH_2_(O)), 4.54 (d, 2H, *J* = 5.6 Hz, CH_2_), 4.64 (s. 2H, CH_2_), 5.20 (t, 1H, *J* = 5.6 Hz, OH), 5.87 (s, 1H, CH), 6.89 (d, 1H, *J* = 7.8 Hz), 7.28 (t, 1H, *J* = 8.0 Hz), 7.84 (m, 2H), 7.94 (d, 1H, *J* = 2.0 Hz), 8.72 (d, 1H, *J* = 2.0 Hz), 9.53 (s, 1H, OH). ^13^C NMR (101 MHz, DMSO-*d*_6_) δ: 47.6 (2 × CH_2_), 49.9 (CH_2_), 55.0 (CH_2_), 66.4 (2 × CH_2_), 114.8 (CH), 117.6 (CH), 119.0 (CH), 123.4 (Cq), 129.3 (CH), 131.8 (CH), 134.4 (Cq), 136.0 (Cq), 139.0 (CH), 145.9 (CH), 147.3 (Cq), 148.6 (Cq), 157.4 (Cq), 158.4 (Cq), 159.2 (Cq). HRMS (EI/MS): *m/z* calculated for C_21_H_21_N_7_O_3_ [M + H]^+^: 420.1706; found 420.1784.

***3-(7-((4-(Fluoromethyl)-1H-1,2,3-triazol-1-yl)methyl)-4-morpholinylpyrido[3,2-d]pyrimidin-2-yl)phenol* (24).** Derivative **18** (35 mg, 0.17 mmol) was subjected to the triazole formation using the same procedure as described for **23**. The crude material was dissolved in dry CH_2_Cl_2_ (4 mL) and the mixture was cooled to 0 °C under inert atmosphere. A solution of DAST (11 µL, 0.083 mmol, 1.1 eq) was added slowly. After stirring at 0 °C, 22 µL of DAST (0.34 mmol, 2.0 eq) was added again. The mixture was stirred at 0 °C for 1 h. Hydrolysis was performed with a saturated solution of NaHCO_3_ (10 mL). The organic layer was extracted with CH_2_Cl_2_ (3 × 10 mL). The organic layer was dried over MgSO_4_ and filtered. The solvent was removed under reduced pressure. The crude product directly underwent the reaction described in general procedure **C** to afford **24** as a yellow solid (44 mg, 62%). Mp: 168–170 °C. IR (Diamond ATR, cm^−1^) ν: 3354, 3046, 1620, 1562, 1506, 1425, 1314, 1266, 1115, 1025, 886, 739, 678. ^1^H NMR (400 MHz, DMSO-*d*_6_) δ: 3.86 (m, 4H, 2 × CH_2_(N)), 4.56 (s, 4H, 2 × CH_2_ (O)), 5.55 (d, 2H, *J* = 48.0 Hz, CH_2_ F), 5.92 (s, 1H, CH), 6.01 (s, 2H, CH_2_), 7.03 (m, 1H), 7.39 (t, 1H, *J* = 8.0 Hz), 7.77 (m, 1H), 7.83 (d, 1H, *J* = 2.0 Hz), 8.09 (s, 1H, CH), 8.56 (d, 1H, *J* = 2.0 Hz), 8.86 (s, 1H, OH). ^13^C NMR (101 MHz, DMSO-*d*_6_) δ: 47.6 (2 × CH_2_), 55.0 (CH_2_), 66.4 (2 × CH_2_), 76.4 (d, *J* = 159.0 Hz, CH_2_), 114.8 (CH), 117.6 (CH), 119.0 (CH), 123.4 (Cq), 129.3 (CH), 131.8 (CH), 134.4 (Cq), 136.0 (Cq), 139.0 (CH), 145.9 (CH), 147.3 (Cq), 148.6 (Cq), 157.4 (Cq), 158.4 (Cq), 159.2 (Cq). ^19^F NMR (376 MHz, DMSO-*d*_6_) δ: −202.6 (CH_2_F). HRMS (EI-MS): *m/z* calculated for C_21_H_21_FN_7_O_2_ [M + H]^+^, 422.1741 found 422.1735.

***2-(2-(3-(Methoxymethoxy)phenyl)-4-morpholinylpyrido[3,2-d]pyrimidin-7-yl)aceto nitrile* (25).** To a solution of *t*-BuOK (108 mg, 0.962 mmol, 1.6 eq.) in dry DME (4 mL) at -50 °C, were added dropwise a solution of TosMIC (141 mg, 0.722 mmol, 1.2 eq.) in dry DME (4 mL) and after 10 min a solution of **8** (230 mg, 0.602 mmol). The reaction was stirred at −50 °C for 40 min, then MeOH (5mL) was added and the solution was refluxed for 1 h. The solvent was removed in vacuo, with the crude product diluted in EtOAc (30 mL). The organic layers were washed with water (2 × 10 mL) and dried over MgSO_4_ and filtered. The solvent was removed under reduced pressure. The crude product was purified by flash chromatography on silica gel (CH_2_Cl_2_/MeOH, 99.4/0.6) to afford **25** as a yellow solid (109 mg, 46%). R_f_ (CH_2_Cl_2_/MeOH, 99.4/0.6): 0.15. Mp: 158-160 °C. IR (ATR diamond, cm^−1^) ν: 2952, 2259, 1600, 1553, 1524, 1502, 1435, 1350, 1271, 1150, 1112, 1078, 1017, 967, 916, 875, 736, 685. ^1^H NMR (400 MHz, CDCl_3_) δ: 3.56 (s, 3H, CH_3_), 3.95 (m, 6H, CH_2_CN and 2 × CH_2_(N)), 4.61 (m, 4H, 2 × CH_2_(O)), 5.31 (s, 2H, CH_2_), 7.20 (ddd, 1H, *J* = 1.1 Hz, *J =* 2.5 Hz, *J* = 8.1 Hz), 7.43 (t, 1H, *J* = 7.9 Hz), 8.16 (m, 3H), 8.62 (d, 1H, *J* = 2.3 Hz, H_6_). ^13^C NMR (101 MHz, CDCl_3_) δ: 21.6 (CH_2_), 48.3 (2 × CH_2_), 56.3 (CH_3_), 67.4 (2 × CH_2_), 94.7 (CH_2_), 116.6 (CH), 118.6 (CH), 122.4 (CH), 129.5 (CH), 129.6 (Cq), 132.9 (Cq), 135.6 (CH), 139.8 (Cq), 145.0 (CH), 148.1 (Cq), 157.6 (Cq), 159.2 (Cq), 160.6 (Cq). HRMS (EI/MS): *m/z* calculated for C_21_H_21_N_5_O_3_ [M + H]^+^: 392.1644; found 392.1718.

***2-(2-(3-Hydroxyphenyl)-4-morpholinylpyrido[3,2-d]pyrimidin-7-yl)acetonitrile* (26).** The reaction was carried out as described in general procedure **C** using **25** (100 mg, 0.255 mmol) to afford **26** as a yellow solid (74 mg, 84%). R_f_ (CH_2_Cl_2_/MeOH, 99/1): 0.12. Mp: 201–203 °C. IR (ATR diamond, cm^−1^) ν: 3402, 2930, 2246, 1616, 1556, 1505, 1439, 1385, 1318, 1245, 1116, 1024, 865, 732. ^1^H NMR (250 MHz, DMSO-*d*_6_) δ: 3.87 (m, 4H, 2 × CH_2_(N)), 4.44 (s, 2H, CH_2_), 4.66 (m, 4H, 2 × CH_2_(O)), 7.08 (d, 1H, *J* = 8.0 Hz), 7.41 (t, 1H, *J* = 7.9 Hz), 8.04-7.76 (m, 2H), 8.46 (s, 1H), 8.82 (d, 1H, *J* = 2.0 Hz, H_6_), 9.85 (s, 1H, OH). ^13^C DEPT NMR (101 MHz, DMSO-*d*_6_) δ: 21.5 (CH_2_), 48.9 (2 × CH_2_), 66.5 (2 × CH_2_), 112.7 (CH), 117.4 (CH), 121.2 (CH), 130.8 (CH), 134.8 (CH). HRMS (EI/MS): *m/z* calculated for C_19_H_18_N_5_O_2_ [M + H]^+^: 348.1382; found 348.1455.

***4-(2-(3-(Methoxymethoxy)phenyl)-7-(2-methoxyvinyl)pyrido[3,2-d]pyrimidin-4-yl)morpholine* (27).** To a solution of methoxymethyltriphenylphosphonium chloride (665 mg, 1.94 mmol, 1.5 eq.) in dry THF (15 mL) at 0 °C was added dropwise a solution of tBuOK (1.94 mL, 1.94 mmol, 1.5 eq.). The mixture was stirred at 0 °C for 1h until a solution of **8** (490 mg, 1.29 mmol) in dry THF (15 mL) was dropwise added. The reaction was stirred at room temperature for 48 h, then H_2_O (30 mL) were added and the crude product diluted in EtOAc (30 mL). The organic layers were extracted with EtOAc (2 × 10 mL) and dried over MgSO_4_ and filtered. The solvent was removed under reduced pressure. The crude product was purified by flash chromatography on silica gel (petroleum ether/EtOAc, 8/2) to afford **27** as a colorless oil containing a mixture of two isomers (327 mg, 80%). IR (Diamond ATR, cm^−1^) ν: 3293, 2856, 1528, 1495, 1442, 1352, 1111, 1021, 861, 739. ^1^H NMR (400 MHz, CDCl_3_) δ: 3.55 (s, 3H, CH_3_), 3.80 (s, 3H, CH_3_ isomer E), 3.92 (s, 2H, CH_3_ isomer Z), 3.94 (m, 4H, 2 × CH_2_(N)), 4.48 (m, 4H, 2 × CH_2_(O)), 5.30 (s, 2H, CH_2_), 5.37 (d, 1H, *J* = 6.8 Hz, CH isomer Z), 5.90 (d, 1H, *J* = 13.0 Hz, CH isomer E), 6.45 (d, 1H, *J* = 6.8 Hz, CH isomer Z), 7.35 (d, 1H, *J* = 13.0 Hz, CH isomer E), 7.41 (t, 1H, *J* = 8.1 Hz), 7.95 (d, 1H, *J* = 1.9 Hz, isomer E), 8.17 (m, 2H), 8.42 (d, 1H, *J* = 1.9 Hz, isomer Z), 8.58 (d, 1H, *J* = 1.9 Hz, isomer E), 8.77 (d, 1H, isomer Z). ^13^C NMR (101 MHz, CDCl_3_) δ: 48.0 (2 × CH_2_), 56.0 (CH_3_), 57.2 (OCH_3_), 61.2 (OCH_3_), 67.3 (2 × CH_2_), 94.5 (2 × CH_2_), 100.9 (CH isomer E), 101.1 (CH isomer Z), 110.4 (Cq), 116.2 (CH), 116.3(CH), 117.9 (CH), 118.0 (CH), 119.8 (CH), 129.9 (CH), 130.4 (CH), 130.8 (Cq), 133.6 (CH), 135.4 (Cq), 135.9 (Cq) 147.8 (Cq), 148.4 (Cq), 148.2 (CH), 146.3 (CH) 152.1 (CH isomer Z), 152.2 (CH isomer E), 157.3 (Cq), 57.4 (Cq), 158.1 (Cq), 159.2 (Cq), 159.6 (Cq), 159.9 (Cq); HRMS (EI-MS): *m/z* calculated for C_20_H_21_N_4_O_3_ [M + H]^+^, 365.1676, found 365.1770.

***2-(2-(3-(Methoxymethoxy)phenyl)-4-morpholinylpyrido[3,2-d]pyrimidin-7-yl) acetaldehyde oxime* (28).** To a solution of **27** (60 mg, 0.178 mmol) in 20 mL THF/H_2_O (3/1) was added mercury acetate (800 mg, 2.51 mmol, 3.0 eq.) at 0 °C. The reaction was stirred for 2 h at 0 °C and brine (5 mL) was added. The organic layers were extracted with EtOAc (3 × 10 mL) and dried over MgSO_4_ and filtered. The solvent was removed under reduced pressure. The crude aldehyde was directly dissolved in CH_2_Cl_2_ (10 mL), hydroxylammonium chloride (112 mg, 1.62 mmol, 2.0 eq.) and next triethylamine (338 µL, 2.43 mmol, 4.0 eq.) were added. The reaction was stirred for 12 h at room temperature, and an aqueous solution of saturated NaHCO_3_ (5 mL) was then added. The organic layers were washed with water (2 × 5 mL) and dried over MgSO_4_ and filtered. The solvent was removed under reduced pressure. The crude product was purified by flash chromatography on silica gel (petroleum ether/EtOAc, 1/1) to afford **28** as yellow solid containing the two stereoisomers (154 mg, 45%). Mp: 154–156 °C. IR (Diamond ATR, cm^−1^) ν: 3200, 2911, 1701, 1640, 1508, 1461, 1426, 1268, 1154, 1116, 1071, 1008, 957, 739. ^1^H NMR (400 MHz, CDCl_3_) δ: 3.52 (s, 3H, CH_3_), 3.72 (d, *J* = 6.1 Hz, CH_2_ for E), 3.92 (m, 6H, CH_2_ for Z et 2 × CH_2_(N)), 4.60 (m, 4H, 2 × CH_2_(O)), 5.28 (s, 2H, CH_2_), 6.88 (t, *J* = 5.5 Hz, 1H, CH_oxime_), 7.18 (m, 1H), 7.40 (t, 1H, *J* = 7.5 Hz), 7.62 (t, *J* = 6.1 Hz, 1H, CH_oxime_), 8.12 (m, 4H,), 8.56 (d, *J* = 2.0 Hz, 1H, isomer *Z*), 8.58 (d, *J* = 2.0 Hz, 1H, isomer *E*), 8.85 (bs, 1H, OH). ^13^C NMR (101 MHz, CDCl_3_) δ: 28.8 (CH_2_ for Z), 33.3 (CH_2_ for E), 48.4 (2 × CH_2_), 55.9 (CH_3_), 66.6 (2 × CH_2_), 94.6 (CH_2_), 115.7 (CH), 118.5 (CH), 122.8 (CH), 129.6 (CH), 131.9 (Cq), 135.1 (CH), 136.2 (Cq), 139.8 (Cq), 146.9 (CH), 147.0 (Cq), 148.3 (CH for E), 148.7 (CH for Z), 157.4 (Cq), 159.2 (Cq), 160.2 (Cq). HRMS (EI-MS): *m/z* calculated for C_21_H_24_N_5_O_4_ [M + H]^+^, 410.1828, found 410.1823.

***4-(2-(3-(Methoxymethoxy)phenyl)-7-((5-(methoxymethyl)isoxazol-3-yl)methyl)pyrido [3,2-d]pyrimidin-4-yl)morpholine* (29).** To a solution of **28** (60 mg, 0.178 mmol) in 4 mL of THF was added methyl propargyl ether (20 µL, 0.22 mmol, 1.1 eq.) and sodium hypochlorite (246 µL, 0.43 mmol, 2.0 eq.) at room temperature. The reaction was stirred for 12 h and H_2_O (5 mL) was added. The organic layers were extracted with EtOAc (3 × 10 mL), dried over MgSO_4_ and filtered. The solvent was removed under reduced pressure. The crude product was purified by flash chromatography on silica gel (EA/EP, 1/1) to afford **29** as a yellow oil (42 mg, 50%). IR (Diamond ATR, cm^−1^) ν: 2923, 2852, 1701, 1640, 1508, 1461, 1426, 1268, 1154, 1116, 1071, 1008, 957, 739; ^1^H NMR (400 MHz, CDCl_3_) δ: 3.41 (s, 3H, CH_3_), 3.52 (s, 3H, CH_3_), 3.92 (m, 4H, 2 × CH_2_(N)), 4.20 (s, 2H, CH_2_), 4.50 (s, 2H, CH_2_), 4.60 (m, 4H, 2 × CH_2_(O)), 5.28 (s, 2H, CH_2_), 6.88 (t, *J* = 5.5 Hz, 1H, CH), 7.18 (m, 1H), 7.40 (t, 1H, *J* = 7.5 Hz), 8.15 (m, 3H), 8.58 (d, *J* = 2.0 Hz, 1H, H_6_), ^13^C NMR (101 MHz, CDCl_3_) δ: 30.0 (CH_2_), 47.7 (2 × CH_2_), 56.2 (CH_3_), 59.1 (CH_3_), 65.7 (CH_2_), 67.4 (2 × CH_2_), 94.8 (CH_2_), 100.1 (Cq), 102.5 (CH), 116.5 (CH), 118.3 (CH), 122.1 (CH), 129.4 (CH), 135.4 (Cq), 136.5 (CH), 136.2 (Cq), 146.9 (CH), 157.2 (Cq), 157.7 (Cq), 159.2 (Cq), 161.2 (Cq), 170.1 (Cq). HRMS (EI-MS): *m/z* calculated for C_25_H_28_N_5_O_5_ [M + H]^+^, 478.2090, found 478.2085.

***3-(7-((5-(Methoxymethyl)isoxazol-3-yl)methyl)-4-morpholinylpyrido[3,2-d]pyrimidin-2-yl)phenol* (30).** The reaction was carried out as described in general procedure **C** using **29** (50 mg, 0.123 mmol) to afford **30** as a yellow solid (52 mg, 98%). Mp: 169–171 °C; IR (Diamond ATR, cm^−1^) υ: 3200, 2911, 1701, 1640, 1508, 1461, 1426, 1268, 1154, 1116, 1071, 1008, 957, 739; ^1^H NMR (400 MHz, DMSO-*d*_6_) δ: 3.52 (s, 3H, CH_3_), 3.90 (m, 4H, 2 × CH_2_(N)), 4.37 (s, 2H, CH_2_), 4.54 (s, 2H, CH_2_), 4.69 (m, 4H, 2 × CH_2_(O)), 6.88 (s, 1H, CH), 7.18 (m, 1H), 7.40 (t, 1H, *J* = 7.5 Hz), 7.80 (m, 2H), 8.38 (m, 1H), 8.88 (d, *J* = 2.0 Hz, 1H), 10.0 (bs, 1H, OH). ^13^C NMR (101 MHz, DMSO-*d*_6_) δ: 30.0 (CH_2_), 47.7 (2 × CH_2_), 58.2 (CH_3_), 66.0 (CH_2_), 68.4 (2 × CH_2_), 104.5 (CH), 116.5 (CH), 118.3 (CH), 120.1 (CH), 130.6 (CH), 135.4 (Cq), 136.2 (CH), 136.5 (Cq), 139.8 (Cq), 146.9 (CH), 147.0 (Cq), 157.1 (Cq), 157.6 (Cq), 159.2 (Cq), 163.1 (Cq), 170.1 (Cq). HRMS (EI-MS): *m/z* calculated for C_23_H_24_N_5_O_4_ [M + H]^+^, 434.1828, found 434.1823.

***2-(3-Hydroxyphenyl)-4-morpholinylpyrido[3,2-d]pyrimidine-7-carbaldehyde oxime* (31).** To a solution of **10** (66 mg, 0.196 mmol), in CH_2_Cl_2_ (10 mL) was added hydroxylammonium chloride (16 mg, 0.236 mmol, 1.2 eq.) and triethylamine (33 µL, 0.236 mmol, 1.2 eq.). The reaction was stirred for 12 h at room temperature, an aqueous solution of saturated NaHCO_3_ (5 mL) was added. The organic layers were washed with water (2 × 5 mL), dried over MgSO_4_ and filtered. The solvent was removed under reduced pressure. The crude product was purified by flash chromatography on silica gel (CH_2_Cl_2_/MeOH, 99/1) to afford **31** as a yellow solid (43 mg, 62%). R_f_ (CH_2_Cl_2_/MeOH, 99/1): 0.10. Mp: 231–233 °C. IR (ATR diamond, cm^−1^) ν: 3277, 2864, 1736, 1561, 1520, 1434, 1356, 1311, 1234, 1107, 972, 739, 678. ^1^H NMR (400 MHz, DMSO-*d*_6_) δ: 3.91–3.69 (m, 4H, 2 × CH_2_(N)), 4.50 (m, 4H, 2 × CH_2_(O)), 6.90 (dd, 1H, *J* = 1.8 Hz, *J* = 7.7 Hz), 7.30 (t, 1H, *J* = 8.0 Hz), 7.99–7.81 (m, 2H), 8.23 (d, 1H, *J* = 2.0 Hz), 8.40 (s, 1H), 9.00 (d, 1H, *J* = 2.0 Hz), 9.54 (d, 1H, *J* = 5.7 Hz, OH), 11.92 (s, 1H, CNH). ^13^C NMR (101 MHz, DMSO-*d*_6_) δ: 47.3 (2 × CH_2_), 66.4 (2 × CH_2_), 114.9 (CH), 117.6 (CH), 119.0 (CH), 129.3 (CH), 132.1 (Cq), 132.3 (Cq), 133.5 (CH), 139.1 (Cq), 143.5 (CH), 145.7 (CH), 147.6 (Cq), 157.4 (Cq), 158.4 (Cq), 159.3 (Cq). HRMS (EI/MS): *m/z* calculated for C_18_H_18_N_5_O_3_ [M + H]^+^: 352.1331; found 352.1407.

***2-(3-Hydroxyphenyl)-4-morpholinylpyrido[3,2-d]pyrimidine-7-carbaldehyde O-methyl oxime* (32).** Compound **32** was obtained as described for **31** starting from **10** (60 mg, 0.178 mmol) and using methoxyammonium chloride (20 mg, 0.232 mmol, 1.2 eq.). The crude product was purified by flash chromatography on silica gel (CH_2_Cl_2_/MeOH, 99/1) to afford **32** as a yellow solid (44 mg, 68%). R_f_ (CH_2_Cl_2_/MeOH, 99/1): 0.28. Mp: 244–246 °C. IR (ATR diamond, cm^−1^) ν: 3040, 2962, 1517, 1442, 1348, 1148, 1115, 1054, 927, 743, 674. ^1^H NMR (400 MHz, DMSO-*d*_6_) δ: 3.83 (m, 4H, 2 × CH_2_(N)), 4.00 (s, 3H, CH_3_), 4.50 (m, 4H, 2 × CH_2_(O)), 6.90 (d, 1H, *J* = 8.1 Hz), 7.30 (t, 1H, *J* = 8.1 Hz), 7.88 (s, 2H), 8.28 (s, 1H), 8.49 (s, 1H, H_oxime_), 8.97 (s, 1H, H_6_), 9.55 (s, 1H, OH). ^13^C NMR (101 MHz, DMSO-*d*_6_) δ: 48.7 (2 × CH_2_), 62.3 (CH_3_), 66.3 (2 × CH_2_), 114.9 (CH), 117.7 (CH), 119.0 (CH), 121.1 (Cq), 129.3 (CH), 131.1 (Cq), 132.4 (Cq), 134.2 (Cq), 143.6 (CH), 146.2 (CH), 157.4 (Cq), 158.4 (Cq), 159.3 (Cq), 164.8 (CH). HRMS (EI/MS): *m/z* calculated for C_19_H_20_N_5_O_3_ [M + H]^+^: 366.1561; found 366.1564.

### 3.2. Molecular Modeling

2D ligand structures were prepared for docking using VSPrep [43]. Tautomer distribution, at pH = 7.4, was evaluated within VSPrep from Chemaxon software [44] and the best tautomer in terms of percentage of the corresponding distribution and visual inspection was retained. A 3D conformation of each compound was finally generated as molecular input for docking.

Protein structure complexes were downloaded from the Protein Data Bank (https://www.rcsb.org/, accessed on 20 August 2018). Chains A and B of PDB ID 4JT6 of the mTOR-PI103 complex structure were separated into two individual structures. Water molecules were removed from PDB ID 4L23, resulting in 4L23 chain A and the corresponding ligand X6K. Complex from PDB ID 4JT6 was separated following the same way, resulting in two complexes of chain A and chain B with their respective X6K ligand. These two chains from PDB ID 4JT6 and the chain from PDB ID 4L23 were superimposed on the kinase library available in the MOE Molecular Suite. [39]

Protein structure complexes were next prepared, with default parameters, within the Protein Preparation Wizard of Maestro from the Schrodinger Molecular Suite [45]. A first step of preprocessing, where bond orders are assigned using the Chemical Components Dictionary database, hydrogen atoms are added, disulfide bonds are created if necessary and ‘het states’ are generated from Epik at pH = 7.0 ± 2.0, is followed by a step of review and modification and finally a step of refinement, where PROPKA with pH = 7.0 is used. Finally, the complex is minimized under a convergence RMSD threshold of 0.30 Å regarding heavy atoms, concluding the preparation. Next, a receptor grid, for each of the three complexes, was generated. Each grid was centered on the co-crystalized ligand PI103 (ligand ID XK6) used as a reference to design the size of the enclosing box. Finally, docking studies were carried out with the Glide module from the Schrodinger Molecular Suite [33].

The docking protocol was validated by redocking the PI103 ligand from a 2D structure. Docking calculations were performed with the XP scoring function with default parameters. Ligand flexibility and ring conformation sampling were taken into account during docking. For each ligand, postdocking minimization was performed on 10 poses. They reproduced complexes with RMSD on heavy atoms of 0.62, 0.22 and 0.27 Å regarding 4JT6.A, 4JT6.B and 4L23, respectively.

### 3.3. Biological Assays

**Cell culture.** Skin normal fibroblastic cells were purchased from Lonza (Basel, Switzerland), HuH7, Caco-2, MDA-MB-231 and Hacat cancer cell lines were obtained from the ECACC collection (Porton Down, UK). Cells were grown at 37 °C, 5% CO_2_ in ECACC recommended media: DMEM for HuH7, MDA-MB-231 and fibroblast, EMEM for CaCo-2 and Hacat. All culture media were supplemented by 10% of FBS, 1% of penicillin-streptomycin and 2 mM glutamine.

**Cytotoxic assay.** Chemicals were solubilized in DMSO at a concentration of 10 mM (stock solution) and diluted in culture medium to the desired final concentrations. The dose effect cytotoxic assays (IC_50_ determination) were performed by increasing the concentrations of each chemical (final well concentrations: 0.1, 0.3, 0.9, 3, 9, and 25 μM). Cells were plated in 96-well plates (4000 cells/well). Twenty-four hours after seeding, cells were exposed to chemicals. After 48h of treatment, cells were washed in PBS and fixed in cooled 90% ethanol/5% acetic acid for 20 min and the nuclei were stained with Hoechst 33,342 (B2261 Sigma). Image acquisition and analysis were performed using a Cellomics ArrayScan VTI/HCS Reader (ThermoScientific). The survival percentages were calculated as the percentage of cell number after compound treatment over cell number after DMSO treatment. The relative IC_50_ were calculated using the curve fitting XLfit 5.5.0.5 (idbs) integrated in Microsoft Excel as an add-on. The 4 Parameter Logistic Model or Sigmoidal Dose-Response Model was used (fit = (A + ((B − A)/(1 + ((C/x)^D))))).

**Western Blot Analysis.** Caco-2 cells were grown in the presence of inhibitors (1 μM) for 48 h. Cellular lysates (PBS/5% SDS lysis buffer containing protease inhibitors and phosphatase inhibitors) were subjected to SDS−polyacrylamide (10%) gel electrophoresis and transferred onto PVDF membranes. Immunoblotting was done using rabbit monoclonal antibodies (Cell Signaling Technology, Ozyme, France) directed against Akt phosphorylated on Thr308 (ref 4056) or using mouse monoclonal antibodies (Sigma–Aldrich Co. ref A1978) directed against Actin. HRP-conjugated rabbit anti-mouse or goat anti-rabbit antibodies (Invitrogen) (1:33 000) were used as secondary antibodies. Immunoreactive bands were detected using the enhanced chemiluminescence reagent (SuperSignal™ West Dura Extended Duration Substrate, Fisher Scientific, France). For quantification, blots were analyzed using Genetools software (Syngene, Frederick, MD, USA).

### 3.4. Kinase Tests

PI3K kinase activity was measured with the Adapta™ assay kit (Invitrogen, PV5099, Life Technologies, Carlsbad, CA, USA) while mTOR kinase activity was measured with the LANCE^TM^ Ultra kit (Perkin Elmer). The Adapta™ assay kit uses a tracer in the form of ADP linked to an acceptor fluorophore (AlexaFluor 647) and an antibody Anti-ADP linked to a donor fluorophore (Eu). Depending on the efficiency of the inhibitor, the level of ADP (which competes with the tracer) will vary, thereby directly affecting the binding of the tracer to the antibody. The TR-FRET signal will evolve accordingly. The working concentrations were 10 μM for ATP (which is lower than the Km) and 50 μM for PIP2 (equal to the Km). The experimental process was the following: the inhibitor solution (2.5 µL) was injected in a plate followed by the addition of 2.5 µL of PI3Kα and ATP/PIP2 (5 µL) solutions. After incubating for an hour at room temperature with no agitation and with the plate sealed, the kinase reaction was stopped by adding the Detection Solution to yield final concentrations of 10 nM for the tracer, 2 nM for the antibody and 30 mM of EDTA (5 µL). All of these compounds were diluted in a Detection Buffer (PV3574, Invitrogen) and the plate was sealed and incubated for the optimal time of 30 min at room temperature without shaking before the reading step. Emission values at 615 nm (corresponding to the donor) and 665 nm (corresponding to the acceptor) were measured and the TR-FRET signal corresponding to the ratio Em 665/Em 615 was calculated.

The LANCE^TM^ Ultra kit (Perkin Elmer) contains a synthetic peptide of 4E-BP1, a natural substrate of mTOR, linked to an acceptor fluorophore (ULight) and an antibody against the phosphorylated form of this peptide linked to a donor fluorophore (Eu). The amount of antibody bound to the peptide is directly related to its rate of phosphorylation. Therefore, the TR-FRET signal decreases with the rate of peptide phosphorylation and a higher concentration of mTOR inhibitor. The experimental process was the following: inhibitor solution (2.5 µL) was injected in a plate followed by the addition of 2.5 µL of mTOR, and ATP/peptide (2.5 µL) solutions. After incubating for two hours at room temperature with no agitation and with the plate sealed, the kinase reaction was stopped by adding 5 μL of a solution at 32 mM EDTA. After 5 min of plate agitation, the detection solution (CR97-100, Perkin-Elmer) containing the antibody (5 μL) was added in each well to yield a final concentration of 2 nM of the antibody. The plate was sealed and incubated for the optimal time of 1h at room temperature without shaking before the assay readout at 665 nm.

Kinase tests using capillary electrophoresis (CE):

The CE system used was a 1600 Hewlett Packard^3D^CE (Agilent, Waldbronn, Germany) equipped with a photodiode array detection system. Agilent software 3D-CE Chemstation (rev B.04.02) was used to pilot the CE system and for signal acquisition. CE analyses were performed in silica capillaries (66 cm total length, 57.5 cm effective length, 50 µm i.d.) purchased from Polymicro Technologies (Phoenix, AZ, USA). New capillaries were conditioned by 1.0 M NaOH for 30 min and water for 5 min. They were then coated by rinsing with cationic PDADMAC solution (0.2% *w*/*v*) [46,47]. The separation was conducted at −30 kV (reverse polarity) and at 30 °C. All rinse cycles were carried out at 4 bars. Between runs, the capillary was flushed for 1 min with water, 2 min with PDADMAC and 3 min with BGE.

All solutions were prepared with pure water, filtered through 0.45 µm PVDF Filter and stored at 4 °C. For PI3K and mTOR assays, the incubation buffer was HEPES/NaOH/MgCl_2_ (25 mM/8.84 mM/5 mM). Its pH was 7.2 and ionic strength was about 60 mM. The BGE was tris/phosphate/MgCl_2_ (117 mM/62.5 mM/5 mM), its pH was 7.2 and ionic strength was 180 mM. All buffers were prepared fresh each day. Their pH was measured with a MeterLab PHM201 Portable pH-Meter (Radiometer Analytical, Villeurbanne, France). Stock solutions of ATP (2 mM) and ADP (2 mM) were prepared in the incubation buffer and diluted to 50 µM. Inhibitor stock solutions were prepared by dissolving 1 mg in 1 mL of incubation buffer and DMSO (<4% *v*/*v*).

In all enzymatic assays, after incubation, the electric field (of −30 kV) was applied to separate the ADP formed during the enzymatic reaction from the other reactants. ADP was detected at 254 nm for quantification. No inhibitor plug was injected to determine the maximum activity of the enzyme; it was replaced by a plug of incubation buffer. Control blank assays were done without injecting the enzyme.

ATP concentrations were set in all assays to be identical to those used in conventional methods (assay kits) for comparison. For IC_50_ determination, enzyme and substrate concentration remained constant whereas at least 11 concentrations of inhibitors were used around the reported IC_50_ value in order to precisely determine IC_50_. Assays were performed in triplicate (*n* = 3). The dose–response curve for enzyme inhibition was carried out by plotting the enzyme activity (%) versus Log [Inhibitor]. GraphPad Prism^®^ (GraphPad Software, Inc., La Jolla, CA, USA) was used for curve-fitting and thus for IC_50_ calculations using the following four parameter logistic equation:

(1) $$\%\;A=\%\;A_{min}+\frac{\%\;A_{max}-\%\;A_{min}}{1+10^{log{(IC}_{50}-[I])\times H}}$$

where % *A* is the enzyme activity (response), % *A_min_* and % *A_max_* are, respectively, the minimal (for maximum inhibition) and the maximal residual enzyme activity, *[I]* is the inhibitor concentration and *H* is the Hill slope factor.

The enzyme activity (%) was defined as the ratio of the corrected peak area (CPA) of ADP formed in the presence and the absence of the inhibitor. The CPA was defined as the peak area divided by the migration time. The CPA of ADP was corrected by removing the CPA obtained for the blank assay.

CE-assay for studying PI3K (α, β, γ and δ) and mTOR kinases was based on using the capillary as a nanoreactor. Transverse diffusion of laminar flow profiles (TDLFP) was used to in-capillary mix reactants and hence to trigger the enzymatic reaction. The injection sequence consisted of introducing the incubation buffer (25 mbar × 50 s) followed by the injection at 50 mbar for 5 s (7.3 nL) of PI3K (2 mg·L^−1^), substrate PIP_2_ (2 g·L^−1^), incubation buffer (or inhibitor in inhibition assays), ATP (50 µM; 0.027 g·L^−1^) and then PI3K (2 mg·L^−1^) a second time. mTOR assays were performed by injecting the incubation buffer (25 mbar × 50 s) followed by the injection at 50 mbar for 5 s of FRAP1/mTOR (10 mg·L^−1^), mTOR substrate (1.25 g·L^−1^), incubation buffer (or inhibitor in inhibition assays), ATP (50 µM) and then FRAP1/mTOR (10 mg·L^−1^) a second time. For both enzymes, the incubation buffer was injected (50 mbar × 30 s) at the end of the injection sequence and reactants were incubated for 10 min. For more details, see [46,47,48,49].

## 4. Conclusions

Trisubstituted 2,4,7-pyrido[3,2-*d*]pyrimidines of type **II** were synthesized using a single trichlorinated platform which was selectively functionalized in each position. Chemical diversity was introduced in C-7 and the designed library was subjected to biological evaluations on enzymes and cell lines. In silico docking studies established the binding mode of most active compounds and confirmed the observed SAR. Addition of a *C*-7 substitution led to improved inhibition compared to *C*-2,4 substituted series and five novel derivatives possessed IC_50_ values between 3 and 13 nM on PI3Kα. Three of them are dual PI3K/mTOR inhibitors, which induced micromolar toxicities on different cancer cell lines. These results proved that a pyridopyrimidine trisubstitution is suitable for developing dual PI3K/mTOR inhibitors with increased effects on different cancer cell lines.

## Figures and Tables

**Table 1:** Evaluation of PI3K inhibition.

Entry	Cpd	Structure	IC_50_ PI3Kα (nM) ^a^	Entry	Cpd	Structure	IC_50_ PI3Kα (nM) ^a^
1	**5**	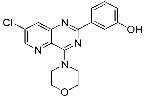	12 ^+/−3^	10	**20**	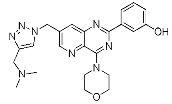	38 ^+/−15^
2	**6**	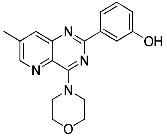	13 ^+/−4^	11	**21**	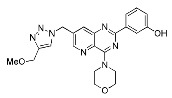	10 ^+/−2^
3	**9**	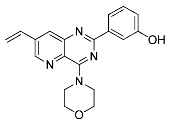	98 ^+/−41^	12	**23**	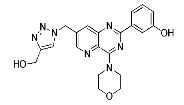	808 ^+/−290^
4	**11**	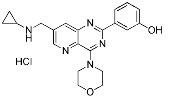	109 ^+/^^−^^9^	13	**24**	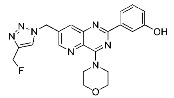	391 ^+/−236^
5	**12**	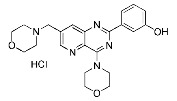	43 ^+/^^−^^13^	14	**26**	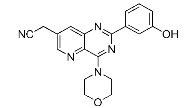	51 ^+/^^−^^18^
6	**13**	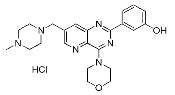	391 ^+/^^−^^260^	15	**30**	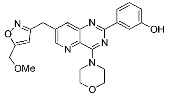	44 ^+/−22^
7	**15**	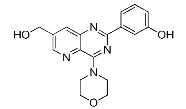	366 ^+/^^−^^201^	16	**31**	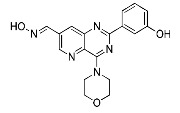	50 ^+/^^−^^21^
8	**16**	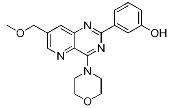	103 ^+/^^−^^54^	17	**32**	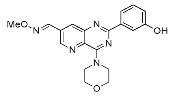	3 ^+/−1^
9	**19**	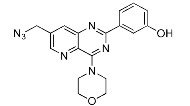	10 ^+/−3^				

^a^ IC_50_ values are presented as the mean of a triplicate experiment.

**Table 2:** Biological evaluation of dual PI3K/mTOR inhibition.

Entry	Number	Structure	IC_50_ PI3Kα (nM) ^a^	IC_50_ mTOR (nM) ^a^	SI (mTOR/PI3Kα Ratio) ^b^	IC_50_ on PI3K Isoforms (nM)
ref	**1**	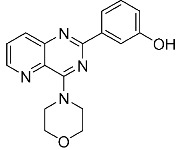	19 ^+/−2^	37 ^+/−12^	1.94	ND
1	**5**	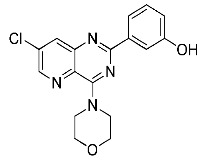	12 ^+/−3^	140 ^+/−28^	11.6	ND
2	**6**	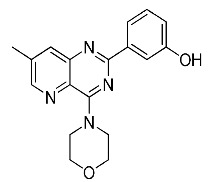	13 ^+/−4^	61 ^+/−18^	4.6	β 450 γ 5500 δ 1400
3	**19**	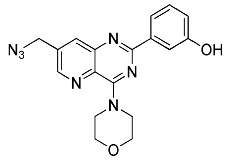	10 ^+/−3^	100 ^+/−20^	10.0	ND
4	**21**	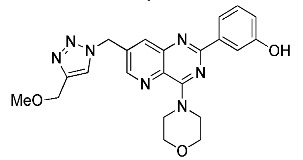	10 ^+/−2^	100 ^+/−31^	10.0	β 50 γ 2000 δ 1200
5	**32**	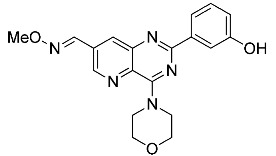	3 ^+/−1^	100 ^+/−26^	33.3	ND

^a^ IC_50_ values are presented as the mean of a triplicate experiment. Standard deviation is indicated in small letters. ^b^ SI selectivity index.

**Table 3:** In cell activity of the best compounds, IC_50_ (μM) ^a^.

Entry	Compounds	Huh-7	Caco-2	MDA-MB 231	Hacat	Fibroblasts
1	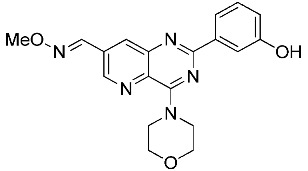 **32**	3 ^+/−2^	3 ^+/−1^	>25	4 ^+/−2^	8 ^+/−2^
2	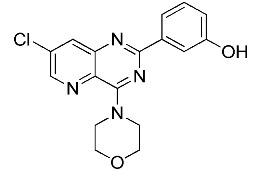 **5**	0.8 ^+/−1^	6 ^+/−3^	>25	4 ^+/−3^	>25
3	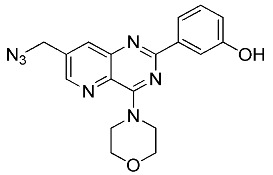 **19**	4 ^+/−1^	2 ^+/−1^	>25	4 ^+/−1^	>25
4	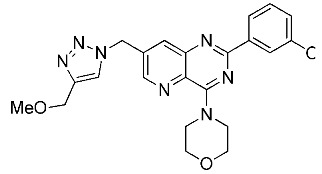 **21**	4 ^+/−1^	1 ^+/−2^	15 ^+/−8^	2 ^+/−3^	>25

^a^ IC_50_ values are presented as the mean of a triplicate experiment. Standard deviation is indicated in small letters.

## Data Availability

Data is contained within the article.

## Sample Availability

Samples of the compounds are not available from the authors.
